# Lunasin alleviates pulmonary inflammation in A549 alveolar epithelial cells and C57BL6/J mice in obese-mimicking conditions

**DOI:** 10.3389/fnut.2026.1732250

**Published:** 2026-02-05

**Authors:** Wan-Sheng Chang, Pei-Ying Huang, Chia-Chien Hsieh

**Affiliations:** Department of Biochemical Science and Technology, College of Life Science, National Taiwan University, Taipei, Taiwan

**Keywords:** immunity, inflammation, lunasin, obesity, pulmonary alveolus

## Abstract

**Background:**

Obesity is accompanied by low-grade and chronic pathological development that can worsen pulmonary inflammation and fibrosis. This study investigated the potential of lunasin, a naturally occurring seed peptide with multiple bioactive properties, to attenuate lung inflammation in A549 pulmonary epithelial cells and in C57BL6/J mice fed with the high-fat diet.

**Methods:**

*In vitro*, palmitic acid (PA) and lipopolysaccharide (LPS) were used to mimic an obese inflammatory microenvironment. The cultured supernatants were collected for cytokine analysis and cells were collected for specific protein analysis. *In vivo*, mice were fed a high-fat (HF) diet or an HF diet supplemented with lunasin-enriched soy protein isolated (HFL) from 6 until 22 weeks of age. The lung and spleen samples were collected for future analysis.

**Results:**

Lunasin inhibited PA- or LPS-induced interleukin (IL)-6, monocyte chemoattractant protein (MCP)-1, and transforming growth factor (TGF)-β secretion. While LPS reduced surfactant protein D (SP-D) expression, lunasin restored SP-D by inhibiting the nuclear factor kappa B (NF-κB) signaling pathway. Additionally, pulmonary fibrosis was induced by TGF-β-induced epithelial-mesenchymal transition (EMT), as indicated by reduced vimentin and preserved E-cadherin expression. However, lunasin did not affect the TGF-β-induced EMT marker in A549 cells. *In vivo*, HFL-fed mice exhibited lower tumor necrosis factor (TNF)-α and TGF-β levels in lung homogenate compared with HF-fed controls. Lunasin supplementation also enhanced the secretion of T helper cell type 1 (Th1) cytokines, including IL-2 and interferon (IFN)-γ, increased the Th1 (IL-2)/Th2 (IL-4) ratio, and reduced the IL-17A level in splenocytes.

**Conclusion:**

In summary, *in vitro*, lunasin attenuated pro-inflammatory cytokines, possibly through enhancing SP-D expression and inhibiting NF-κB signaling in A549 cells. *In vivo*, dietary lunasin supplementation reduced pulmonary inflammation and modulated splenic cytokine balance. This study reveals for the first time that lunasin is a promising candidate for mitigating obesity-related pulmonary inflammation.

## 1 Introduction

Over the last decade, pulmonary disease has emerged as one of the most prevalent acute and chronic conditions, leading to huge healthcare costs. Obesity contributes significantly to this burden linked to pulmonary inflammation, fibrosis, and worsened respiratory outcomes (1). In morbid obesity, dysfunctional adipose tissue sustains systemic low-grade inflammation, further impairing pulmonary immune defense and heightening susceptibility to respiratory complications, such as airway hyper-responsiveness. Beyond inflammation, excessive fat accumulation also imposes mechanical constraints on breathing, increasing ventilator demand and the risk of respiratory diseases (1, 2).

During adiposity, adipocytes function as an endocrine organ, secreting adipokines, cytokines, and chemokines that recruit multiple immune cells (3). Adipocytes release higher levels of pro-inflammatory cytokines, including tumor necrosis factor alpha (TNF-α), interleukin (IL)-6, leptin, and monocyte chemoattractant protein (MCP)-1, and, combined with reduced anti-inflammatory adiponectin, foster a pro-inflammatory microenvironment (4). This imbalance microenvironment impairs lung function, accelerates the progression of various pulmonary injuries and diseases, including airway hyperresponsiveness, and increases susceptibility to conditions such as asthma, acute respiratory distress syndrome (ARDS), chronic obstructive pulmonary disease (COPD) (4), obstructive sleep apnea (3), and severe coronavirus disease (COVID)-19 (5). Pulmonary fibrosis is strongly associated with chronic lung inflammation, resulting in progressive scarring, structural remodeling, and impaired respiratory function. It has been demonstrated that there is a link between lung fibrosis and diet-induced obesity (6), primarily driven by the inflammatory mediators TNF-α and MCP-1 and transforming growth factor beta (TGF-β), contributing to obesity-related lung fibrosis and worsening outcomes in idiopathic pulmonary fibrosis patients. Inflamed lungs release these mediators locally from multiple cell types, shifting the tissue environment toward profibrotic remodeling (4).

Regarding pulmonary immune defense, obese individuals with impaired immune defense were vulnerable during the COVID-19 pandemic in 2020 (7). Systematic reviews and meta-analyses consistently report that obesity increases severity, adverse outcomes, and mortality in COVID-19 (8). Therefore, obesity has been identified as a significant risk factor for poor outcomes in this infectious disease.

The key component of pulmonary defense is the surfactant system, which coats the alveoli to reduce surface tension and prevent alveolar collapse (9). Pulmonary surfactants, secreted by alveolar type (AT) II cells, are amphiphilic molecules composed of phospholipids, cholesterol, and surfactant proteins (SPs), while SP-A and SP-D participate in innate immune defense and facilitate pathogen clearance. Additionally, SP-B and SP-C are involved in surfactant synthesis, structure, and spread during breathing (10). Therefore, surfactants are central in maintaining lung function and respiratory health.

Dietary strategies and lifestyle modifications are associated with mitigating obesity and reducing the risk of respiratory diseases. Among these approaches, food-derived bioactive compounds have attracted increasing scientific interest as potential adjunct therapies in lung diseases (11, 12). Moreover, dietary factors directly influence the gut microbiota, metabolites, and the gut environment, and these changes can affect distant organs through circulation. Gut microbes, metabolites, and immune cells are trained in this environment and travel to the lungs and regulate airway immunity (13). The dietary bioactive compound-microbiota interactions are well-established. Dietary fiber and its derived short-chain fatty acids can modulate several immune cells, mediate gut-lung crosstalk involved in chronic pulmonary disease, and exert anti-inflammatory properties (14). Polyphenols and polysaccharides also alleviate inflammatory bowel disease and increase the abundance of beneficial microbes (15). Recently, soy-derived hydrolyzed peptides intervention in a high-fat diet significantly reduced serum leptin and insulin resistance, decreased intestinal inflammation, and regulated intestinal barrier in mice (16).

Based on the evidence, there is growing interest in exploring natural bioactive compounds to alleviate pulmonary diseases. Lunasin, a seed peptide found in legumes and cereals, exhibits diverse biological activities, including anticancer, anti-inflammatory, antioxidant, lipid-regulating, glycemic homeostasis, and immuno-modulatory properties (17–19). For these reasons, lunasin holds promise as an agent for addressing obesity-related comorbidities. Thus, investigating its effects on pulmonary alveoli under obesity-associated conditions may provide insights into its ability to mitigate lung inflammation and fibrosis.

Obesity is a chronic disease that impairs multiple organ systems, including the lungs. As such, effective therapeutic strategies targeting inflammation and fibrosis are urgently needed. There is still a lack of studies examining the effects of lunasin in alveolar type II cells under obesogenic conditions. Therefore, we investigated the effects of lunasin on A549 pulmonary epithelial cells under obesity-mimicking conditions, focusing on pro-inflammatory mediators, signaling pathways, and epithelial-mesenchymal transition (EMT) markers. To validate these findings, we performed *in vivo* experiments in mice fed with a high-fat diet supplemented with lunasin, to order to assess immune responses in the lungs and spleen.

## 2 Materials and methods

### 2.1 Cell culture

A549 pulmonary epithelial cells were purchased from the Bioresource Collection and Research Center (#60074, BCRC, Hsinchu, Taiwan). The complete medium for cells was Ham's F-12k medium (ACE Biolabs, Taoyuan, Taiwan), containing 7 mm glucose and 10% fetal bovine serum (FBS; Gibco, Waltham, Massachusetts, USA). Cells were maintained at 37 °C with 5% CO_2_ and humidification.

### 2.2 Obesity-related conditions

To generate obese microenvironments *in vitro*, A549 cells were treated with palmitic acid (PA; Sigma, St. Louis, USA) in accordance with obesity-related conditions (18). PA was prepared in absolute ethanol and then conjugated with 1% bovine serum albumin (BSA; Sigma) in Ham's F-12k media in a 37 °C incubator for 2 h. After sterilization using a filter, the PA stock was stored at −20 °C. The working concentration of PA was 500 μm. Additionally, lipopolysaccharide (LPS; Sigma) was used as another model of obesity, as a higher level of endotoxin has been reported in obesity (20), and it generally stimulates acute inflammation (19). The working concentration of LPS was 10 μg/ml. Moreover, TGF-β (PeproTech, Thermo Fisher Scientific, Cranbury, New Jersey, USA) was used to induce lung fibrosis *in vitro*, with a working concentration of 10 μg/ml. LPS and TGF-β were prepared and stored at −20 °C for future use. The lunasin peptide was chemical synthesized with a purity more than 95% (Synpeptide Co., Ltd., Shanghai, China). The working concentrations of lunasin were 5 and 50 μm. Cells were cultured with obesity-related conditions, with or without lunasin for 24 h.

### 2.3 Cell viability

A549 cells were treated with serial doses of lunasin, PA, or LPS for 48 h. After removing the media, the cells were incubated in 0.5 mg/ml 3-(4, 5-Dimethylthiazol-2-yl)-2, 5-diphenyltetrazolium bromide (MTT, Sigma) in DMEM at 37 °C for 3 h. After aspirating, DMSO was added, and formazan crystals were solubilized on the shaker. The absorbance was determined at 540 nm using a spectrophotometer (Molecular Devices, CA, USA). Cell viability was calculated using the formula and is presented as percentages.


$$\text{Cell viability}\left(\%\right)=\;\left[\frac{(\text{OD sample }-\text{ OD blank}}{\left(\text{OD control OD blank}\right)}\right]\;\times 100$$

### 2.4 Assessment of cytokine secretion using enzyme-linked immunosorbent assay (ELISA)

A549 cells were cultured in the presence or absence of 500 μm PA or 10 μg/ml LPS and treated with lunasin for 48 h. Then, supernatants were collected for cytokine analysis. IL-6, MCP-1, and TGF-β secretions were analyzed via ELISA, in accordance with the manufacturer's protocol (BioLegend, San Diego, CA, USA) and a previous study (19). Briefly, the plates were coated with capture antibodies overnight, then blocked and subsequently incubated with standards or samples. After washing, the plates were processed with detection antibodies, horseradish peroxidase-conjugated streptavidin, and the substrate. Absorbance was measured using a spectrophotometer (Molecular Devices, CA, USA), and the concentration was calculated based on the standard curve.

### 2.5 Migration assay

Cells were seeded at 1.1 × 10^5^ cells/well in 24-well plates overnight until cell confluence reached 80%. Cells were pre-treated with lunasin for 4 h, and cellular monolayers were scratched using a 1,000 μl pipette tip. Then, the cultured media were replaced with either 0.5% FBS/F-12 medium, 5 ng/ml TGF-β, or 100 ng/ml recombinant mouse leptin (R&D, Minneapolis, MN, USA), with or without lunasin, for 48 h. Imaging was performed 0, 24, and 48 h post-scratch using a microscope at 100 × magnification (Leica). Migrated areas were quantified using Image J, and the results are presented as the Migration index. Additionally, the healing area under the curve (AUC) was calculated according to biopharmaceutical references.


$$\begin{array}{l} \text{Migration index }\left(\%\right)=\\ \;\left[\frac{\left(\text{initial scratched area}-\text{final scratched area}\right)}{\text{initial scratched area}}\right]\;\times 100 \end{array}$$

### 2.6 Western blot analysis

Cells were pre-treated with lunasin for 4 h and then cultured with 500 μm PA or 10 μg/ml LPS with or without lunasin for 24 h. Cells were then collected and extracted using radio immunoprecipitation assay (RIPA) buffer (Bioman, Taiwan) containing protease inhibitors (Targetmol, Boston, USA) and phosphatase inhibitors (Targetmol). Cell lysates were adjusted for protein loading according to a BCA protein assay (T-PRO, Taiwan) and denatured in a 100 °C water bath for 10 min. Then, samples were subjected to sodium dodecyl sulfate-polyacrylamide gel electrophoresis (SDS–PAGE) and electrotransferred to a PVDF membrane. Briefly, membranes were blocked in gelatin-NET buffer and then incubated with primary antibodies (Ab), containing anti-surfactant protein (SP)-D (abcam), anti-phospho-nuclear factor kappa B (NF-κB) p65 (phosphor Ser536; Santa Cruz, California, USA), anti-NF-κB p65 (Santa Cruz), and anti-GAPDH (abcam, Cambridge, UK), as well as secondary peroxidase AffiniPure goat anti-mouse or anti-rabbit antibody (Jackson, Pennsylvania, USA). After washing, targeted proteins were visualized using a LumiLong with chemiluminescence detection (Tprobio, Taiwan). The image was quantified using ImageJ (version 1.51j8; SciJaVa Common Library).

### 2.7 EMT immunofluorescence

Cells were set at 2.5 × 10^4^ cells/ml in a μ-Slide eight Well (ibidi, Gräfelfing, Germany) and pre-treated with lunasin for 4 h. The cells were then cultured with 5 ng/ml TGF-β or 100 ng/ml leptin, with or without lunasin, for 48 h. After washing, cells were fixed in 4% paraformaldehyde (Sigma) for 10 min and permeabilized in 0.2% Triton-X 100 (Sigma) for 15 min. After washing, the slides were blocked with 5% normal serum (Jackson ImmunoResearch, West Grove, Pennsylvania, USA) in 0.3% Triton-X 100 and processed with E-cadherin (Cell signaling, Danvers, Massachusetts, USA) and vimentin (BioLegend) primary antibodies and CF™488A-conjugated and CF^TM^ 594-conjugated fluorescein secondary antibodies (Sigma). Nuclei were stained with the NucBlue™ fixed cell ready Probes™ reagent (Invitrogen™, California, USA). Images were taken at 200 × magnification using a fluorescence microscope (Leica, Wetzlar, Germany) and quantified using ImageJ (1.51j8; SciJAVA Common Library) by counting three random fields under a microscope.

### 2.8 Experimental animals and diet

Male C57BL/6JNarl mice were purchased from the National Laboratory Animal Center (Taipei, Taiwan). The experimental animal protocol was approved (#111-00118, Institutional Animal Care and Use Committee, National Taiwan University, Taipei, Taiwan), and animal care was conducted in accordance with animal welfare, ethical guidelines, and the 3R, as outlined in the National Research Council's Guide for the Care and Use of Laboratory Animals. Mice were housed in groups of three in polycarbonate cages measuring 30 × 20 × 15 cm under a 12-h light/dark cycle, with the light period occurring from 7:00 a.m. to 7:00 p.m. The temperature was maintained at 24 °C ± 2 °C and the humidity was kept at 60 ± 10%. After a one-week acclimatization period, 6-week-old mice were divided into two groups: a high-fat and high-fructose (HF) group and an HF diet was added the lunasin-enriched soy protein isolated (Archer Daniels Midland, Chicago, USA), at a lunasin concentration of 483 mg/kg in the diet (HFL; *n* = 6/group). The table of dietary composition is included in Supplementary material 1, and the actual lunasin content was specified in the footnote. The lunasin-enriched soy protein isolated was prepared and replaced the part of casein in the diet. A sample size of six mice per group was selected based on a similar high-fat diet study (21). Ethical constraints were also considered to meet scientific requirements, while following the 3Rs principles, and randomization to minimize variability and ensure reliable data (22). In 1999, the FDA approved a health claim stating that 25 g/day of soy protein may reduce the risk of coronary heart disease (23). The lunasin concentration in HFL was equivalent twice the FDA-recommended daily intake of soy protein. The experimental HF diet, in which 45% calories came from fat and 27% calories came from fructose, mimics diet-induced obesity. Mice were fed the experimental diets *ad libitum* from 6 weeks old until 22 weeks old. The animals were monitored daily for overall health, activity levels, and food intake. Body weight was measured weekly to assess their wellbeing and identify any potential distress. At the end of the experiment, mice were humanely euthanized in accordance with approved guidelines to minimize pain and distress. Euthanasia was performed by CO_2_ inhalation at a flow rate of 30% of the chamber volume per minute, and CO_2_ flow was maintained for at least 1 min after respiratory arrest. After exposure, mice that were dead or deeply anesthetized, followed by cervical dislocation to ensure irreversible death. Lung and spleen samples were collected and stored at −80°C for the future analysis.

### 2.9 Cytokine secretion in lung tissue and homogenate

Lung tissues were separated for two experiments. Firstly, lung tissue was cut into 0.1 mg pieces and added to RIPA buffer at a concentration of 0.1 mg/ml to facilitate homogenization. The homogenate was then centrifuged at 13,000 × g at 4 °C for 20 min, and the supernatant was collected for cytokine analysis. In another experiment, lung tissue was cut into 0.05 mg and cultured in 1 ml of Roswell Park Memorial Institute (RPMI)-1640 medium (Gibco, Waltham, Massachusetts, USA) with 1% fetal bovine serum (Gibco) in 24-well plates in the presence or absence of 1 μg/ml LPS (Sigma) for pro-inflammatory stimulation. After 24 h of culturing, the supernatants were harvested for cytokine analysis. The method was based on, and modified according to a previous study (24). Cytokine levels were adjusted based on the protein concentration in individual lung tissues. The cytokines IL-6, TNF-α, and TGF-β were assayed according to the manufacturer's protocols (BioLegend).

### 2.10 Cytokine secretion in splenocytes

The spleen was collected, ground up, and processed with erythrocyte lysis and ammonia chloride-tris buffer to release signal cells. After washing with Hank's balanced saline solution (HBSS; Sigma), splenocytes were placed at 5 × 10^6^ cells/ml RPMI-1640 medium (Gibco) with 10% mouse serum replacement (Protide Pharmaceuticals, Illinois, USA) in 48-well plates and activated using 10 μg/ml LPS and concanavalin A (ConA, Sigma) for 48 h. Then, supernatants were collected for cytokine analysis. The cytokines under study were analyzed according to the manufacturer's protocols and included IL-2, IL-4, IL-6, IL-17A, TNF-α, and interferon (IFN)-γ (BioLegend).

### 2.11 Statistical analysis

All data used for this study was available in Supplementary material 3. In the cellular study, data were obtained from at least three independent experiments. In the animal study, data from five to six mice were presented as the mean ± standard error of the mean (SEM). Statistical analysis was performed via Student's *t*-test using Statistical Product and Service Solutions (SPSS, version 25; Armonk, NY, USA). A statistically significant difference was considered for values of *P* < 0.05.

## 3 Results

### 3.1 Use of PA and LPS to mimic obese conditions in A549 cells

**Figure 1:**
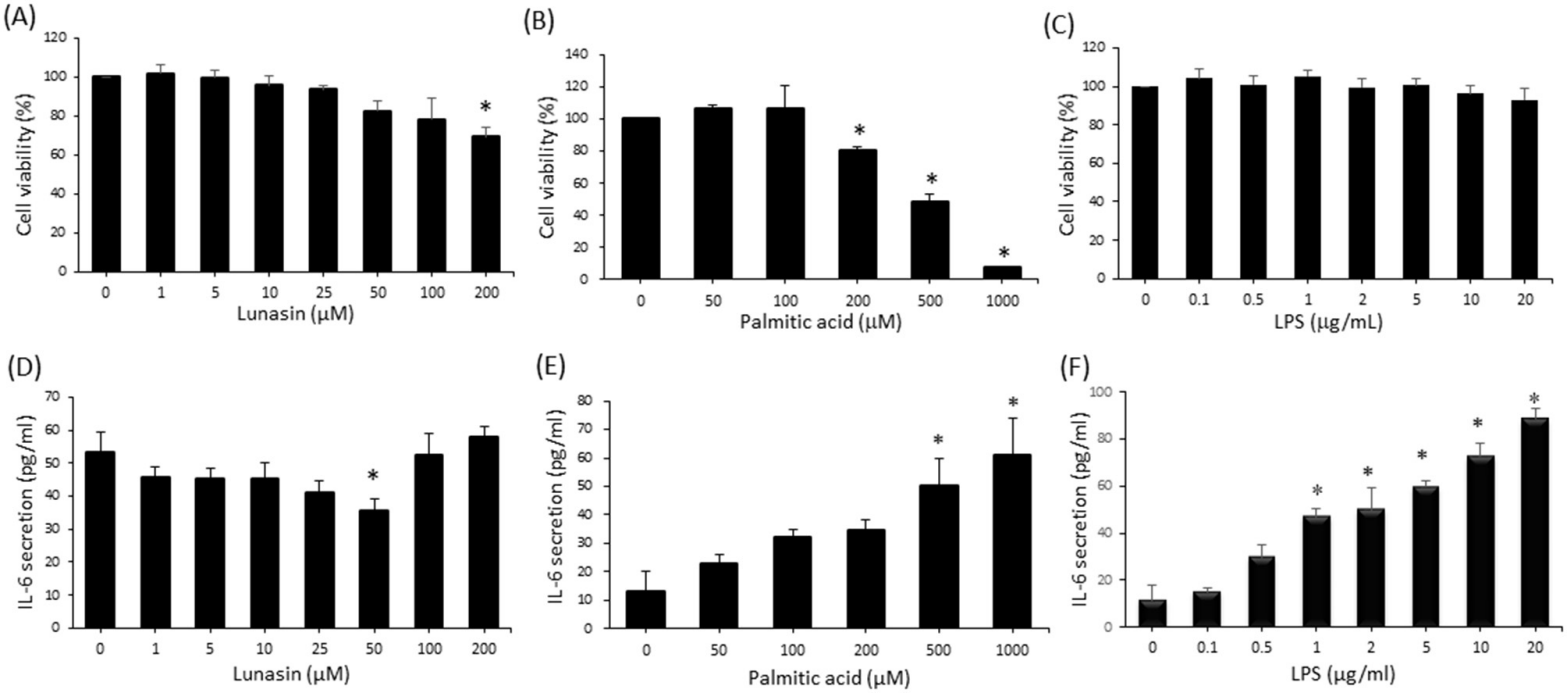
The effect of lunasin and obesity-related factors on cell viability and inflammatory cytokine IL-6 secretion in A549 cells. Cells were treated with various doses of **(A)** lunasin (0–200 μm), **(B)** palmitic acid (PA, 0–1,000 μm), and **(C)** lipopolysaccharide (LPS, 0–20 μg/ml) for 48 h, and cell viability was determined via MTT assay. Cultured supernatants were collected to assess IL-6 secretion via ELISA analysis in **(D)** lunasin, **(E)** PA, and **(F)** LPS treatments. Data are presented as the mean ± SEM of at least three independent experiments. Statistical analysis was based on Student's *t*-test. *P < 0.05 for treatment vs. the control group.

A549 cells were treated with serial doses of lunasin, PA, and LPS for 24 h, and cell viability was detected via MTT (Figures 1A–C). Cultured supernatants were analyzed for the pro-inflammatory cytokine IL-6 (Figures 1D–F) to confirm the toxicity and stimulatory effects of the reagents. Treatments with lunasin at 1 to 100 μm and LPS at 0.1 to 20 μg/ml did not affect A549 cell viability, suggesting that these treatments did not exhibit toxicity; however, PA at concentrations greater than 200 μm significantly reduced cell viability. Regarding the pro-inflammatory effect, A549 cells treated with PA at concentrations higher than 500 μm and LPS at concentrations higher than 1 μg/ml showed a significant increase in IL-6 production, but 50 μm lunasin reduced IL-6 secretion (*P* < 0.05). Therefore, in future experiments, treatment doses were selected at 5 and 50 μm of lunasin, corresponding to low and high doses, and are also similar to previous studies on its anti-inflammatory properties (17, 19, 25). We used 500 μm of PA and 10 μg/ml of LPS to mimic obese conditions, referring to these concentrations, which represent an inflammatory response characterized by an increase in IL-6. Additionally, the selected concentrations of PA and LPS were similar to those in other related studies (26, 27).

### 3.2 Lunasin inhibited inflammation under obese microenvironments in A549 cells

**Figure 2:**
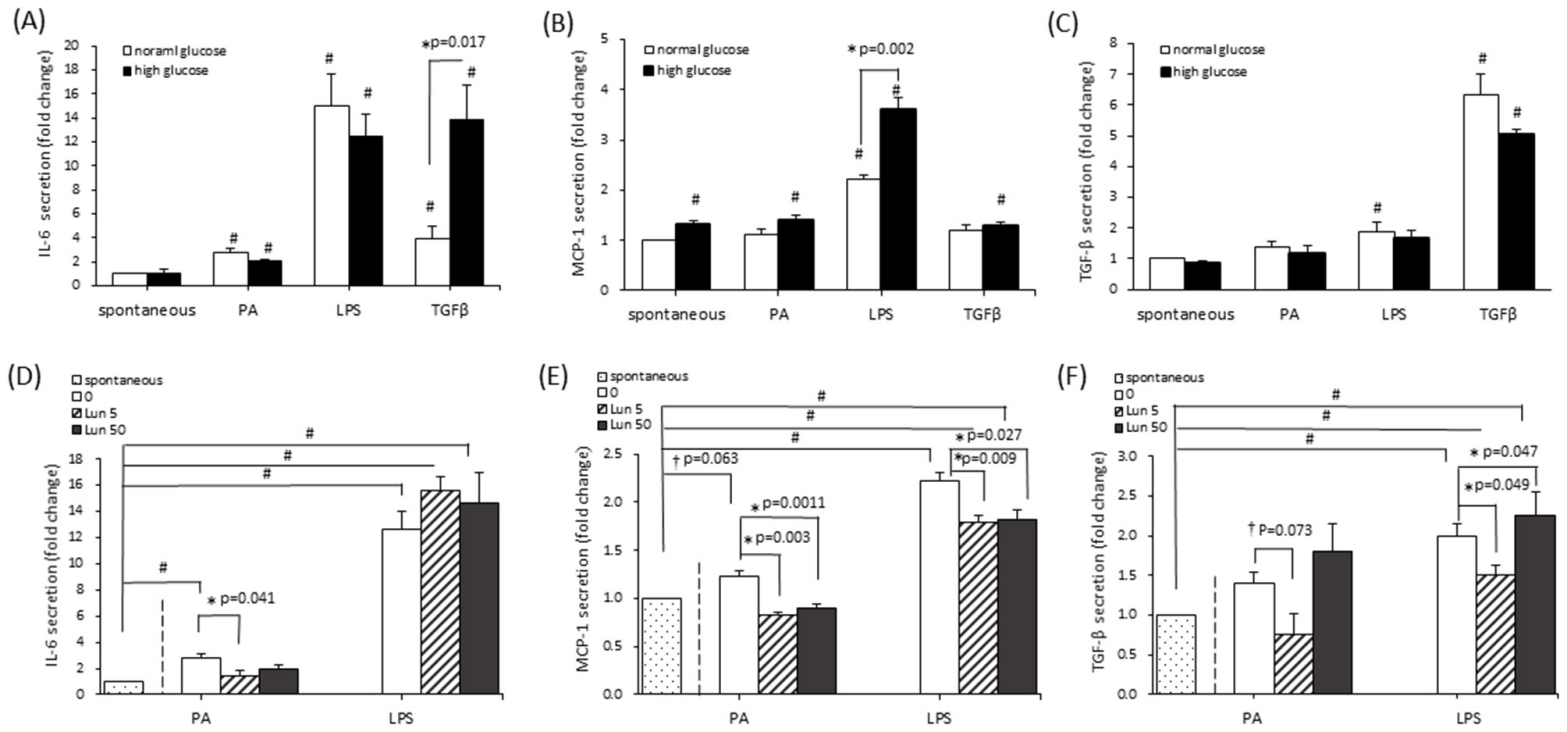
The effect of lunasin on inflammatory cytokine secretion in A549 cells cultured in obese microenvironments. Cells were cultured in normal-glucose (7 mm) medium or high-glucose (35 mm) medium, and supernatants were collected for the analysis of **(A)** IL-6, **(B)** MCP-1, and **(C)** TGF-β production. Additionally, cells were treated with lunasin and stimulated via PA and LPS in normal-glucose media for **(D)** IL-6 and **(E)** MCP-1 and in high-glucose media for **(F)** TGF-β productions. The supernatants were analyzed using ELISA and normalized based on individual cell numbers. Data were calculated by comparing the control and are presented as fold values relative to the control (fold change), with mean ± SEM from at least three independent experiments. Statistical analysis was based on Student's *t*-tests. ^#^*P* < 0.05 treatment vs. the spontaneous group; *P < 0.05 treatment vs. the model control;^†^*P* < 0.1 treatment vs. model control. Lun, lunasin; PA, palmitic acid; LPS, lipopolysaccharide; TGF-β, transforming growth factor β.

To explore whether lunasin influences inflammatory mediators under obese conditions, cells were cultured in high-glucose, PA or LPS conditions to mimic obese micro-environments and treated with TGF-β to induce fibrosis. Because long-term obesity is often accompanied by insulin resistant and hyperglycemia (28). Therefore, a high-glucose condition was also included in this experimental design. The cultured supernatant was analyzed for the production of the cytokines IL-6, MCP-1, and TGF-β via ELISA. Firstly, to confirm whether glucose levels influenced cellular inflammation, cells were cultured in normal-glucose (7 mm) or high-glucose (35 mm) media and challenged with PA, LPS, or TGF-β at the same time. Pro-inflammatory IL-6 and MCP-1 cytokines were significantly increased under obesity-related challenge (Figures 2A, B). In high-glucose media, IL-6 and MCP-1 levels were increased through TGF-β and LPS stimulation, respectively. While TGF-β secretion was induced by LPS stimulation, it was not affected by glucose levels (Figure 2C). Therefore, the inflammatory cytokines IL-6 and MCP-1 were affected by high glucose, but this was not observed for the fibrotic mediator TGF-β.

Lunasin treatment reduced IL-6 and MCP-1 production induced by PA and suppressed MCP-1 secretion triggered by LPS in A549 cells cultured in normal-glucose media (Figures 2D, E). At a low dose of 5 μm, lunasin also decreased TGF-β production in response to LPS stimulation and slightly inhibited PA-induced TGF-β compared with the control group in cells cultured in high-glucose media (*P* = 0.073; Figure 2F). Collectively, lunasin significantly reduced the production of the pro-inflammatory mediators IL-6 and MCP-1, as well as the profibrotic mediator TGF-β, in obese-mimicking microenvironments, independent of glucose levels. Therefore, subsequent experiments were conducted using normal glucose combined with PA or LPS. Regarding the validity of the lunasin dosage, a low concentration of 5 μm lunasin markedly reduced IL-6, MCP-1, and TGF-β production, indicating that this level was optimal for achieving anti-inflammatory activity in A549 cells. However, a higher concentration of 50 μm slightly reduced cell viability, although the effect was not significant.

### 3.3 Lunasin inhibited the SP-D through the NF-kB signaling pathway

**Figure 3:**
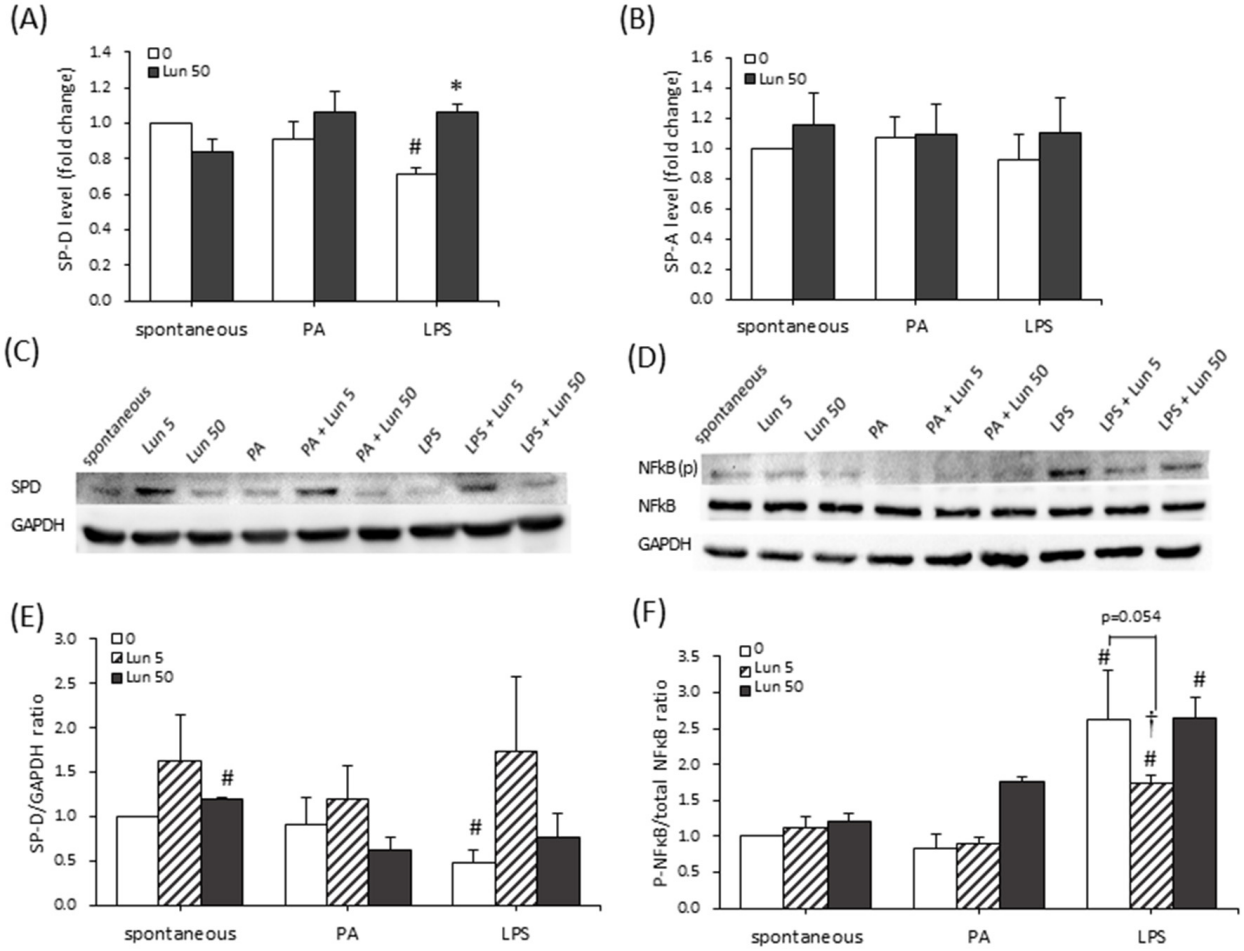
Effect of lunasin on surfactant protein expression in A549 cells cultured in obese micro-environments. Cells were treated with lunasin in the presence or absence of PA or LPS conditions. Then, cells were collected for **(A)** SP-D and **(B)** SP-A analysis via ELISA. Additionally, cells were treated and collected for the assessment of **(C)** SP-D protein and **(D)** Total NF-κB p65 and phosphorylated (p)-NF-κB p65 expression using Western blotting. GAPDH was used as a loading control. Protein expression was quantified in **(E)** SP-D and **(F)** p-NF-κB p65 expression using Image J software. Data are presented as the mean ± SEM of three independent experiments. Statistical analysis was based on an independent-samples *t*-test. ^#^*P* < 0.05 treatment vs. spontaneous group; *P < 0.05 treatment vs. model control;^†^*P* < 0.1 treatment vs. model control.

To investigate whether lunasin modulates SPs and provides protective effects, A549 cells were treated with lunasin under spontaneous, PA, or LPS conditions. The expression of SP-A, SP-D, and their possible signaling molecule NF-κB was detected (Figure 3). The SP-D level in A549 cells was inhibited by LPS stimulation, whereas it was significantly restored under lunasin treatment compared with LPS only, as determined via ELISA (*P* < 0.05; Figure 3A); however, this effect was not observed for SP-A (Figure 3B). According to Western blot analysis, 50 μm lunasin consistently increased SP-D production under spontaneous conditions. Additionally, LPS reduced the level of SP-D protein, while 5 μm lunasin tended to increase its expression under LPS stimulation, although without a significant difference (Figures 3C, E). The discrepancy regarding effective doses between ELISA and Western blot analysis likely reflects differences in the detection of secreted vs. intracellular proteins and the duration of treatment.

In an advanced investigation, the molecular signaling of SP-D following lunasin treatment, including NF-κB and its phosphorylation level, was analyzed and quantified (Figures 3D, F). LPS challenge significantly enhanced the phosphorylation of NF-κB, and treatment with 5 μm lunasin reduced this phosphorylation (*P* = 0.054), but no such observation was made for the PA model. Based on this evidence, lunasin significantly enhanced SP-D protein expression by suppressing NF-κB phosphorylation, which subsequently inhibited the production of inflammatory cytokines in A549 cells under obesity-related conditions.

### 3.4 Lunasin did not affect migration or EMT expression in A549 cells

**Figure 4:**
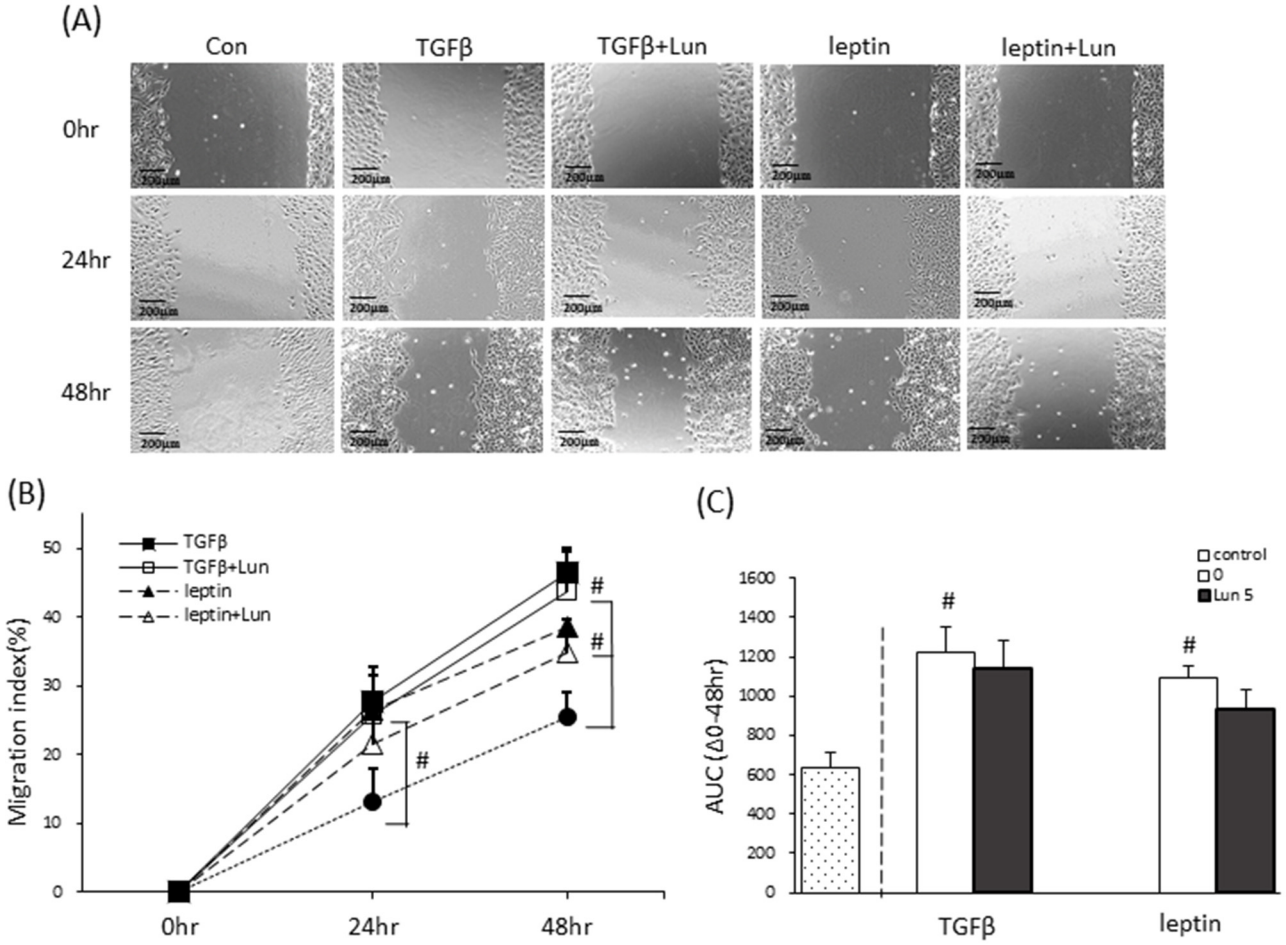
Effect of lunasin on the migration of A549 cells cultured in obese microenvironments. Cell layers were scratched and treated with lunasin in the presence or absence of 5 ng/ml TGF-β or 100 ng/ml leptin for 48 h. The wound healing assay was used to determine the migration of cells. **(A)** Images were taken using a light microscope (×100 magnification). **(B)** The migration index was quantified using Image J. **(C)** The AUC was calculated as the area under the line. Data are presented as the mean ± SEM of five independent experiments. Statistical analysis was based on an independent-samples *t*-test. ^#^*P* < 0.05 treatment vs. spontaneous group.

To assess whether lunasin interferes with fibrosis in A549 cells, wound-healing assays were performed under TGF-β and leptin stimulation to mimic an obese microenvironment. PA treatment did not influence cell migration; thus, it was excluded as a fibrosis model, and the data are not shown. The wound healing assay was analyzed to assess cell migration and also as an indicator of cell fibrosis. Confluent cell monolayers were scraped, and the area of wound closure was evaluated at 0, 24, and 48 h (Figure 4A). The cell layer of the control group showed a significant distance in the scraping. Compared with controls, TGF-β and leptin significantly promoted cell migration, as reflected in the migration index and AUC (Figures 4B, C). However, lunasin treatment did not alter wound healing under either TGF-β or leptin stimulation after scraping compared with the control group.

**Figure 5:**
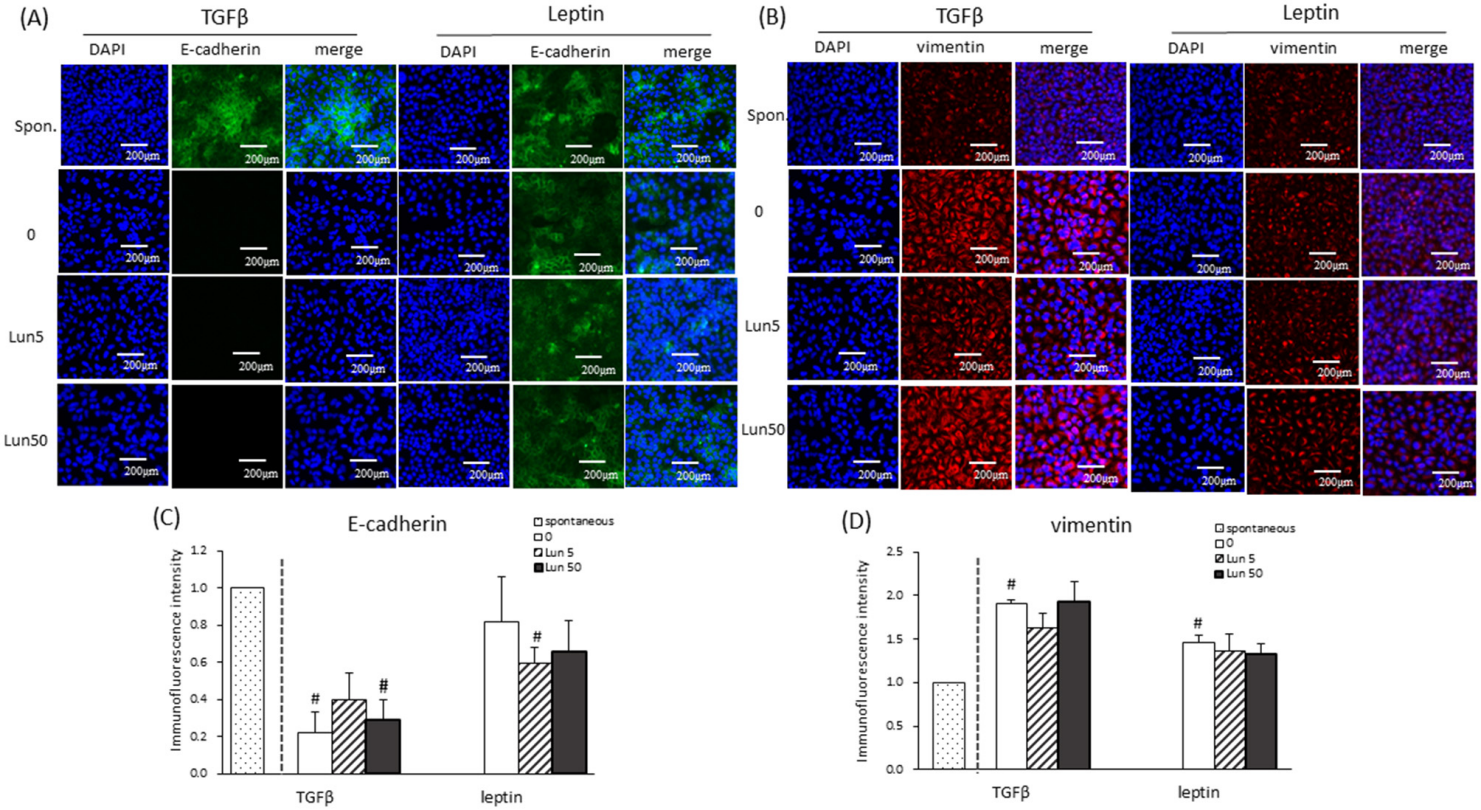
Effect of lunasin on epithelial-mesenchymal transition marker expression in A549 cells cultured in obese microenvironments. Cells were treated with lunasin in the presence or absence of TGF-β or leptin for 48 h. E-cadherin and vimentin protein expression was determined based on immunofluorescence. **(A)** E-cadherin expression was visualized using green immunofluorescence, **(B)** vimentin expression was visualized using red immunofluorescence, and nuclei were visualized using DAPI (blue). Images were observed at a magnification × 200 under a fluorescence microscope. **(C, D)** The images were quantified using Image J. The mean fluorescence intensity was calculated and compared with the spontaneous group as a fold value in relation to the control. Each bar represents the mean ± SEM of at least three independent experiments. Statistical analysis was based on an independent-samples *t*-test. ^#^*P* < 0.05 treatment vs. spontaneous group.

To further evaluate the role of lunasin in fibrosis, EMT markers were examined in A549 cells. Vimentin (red), a mesenchymal marker, is highly expressed during EMT, whereas E-cadherin (green), an epithelium marker, is abundant in normal epithelial cells, as shown via immunofluorescence (Figures 5A, B). The quantification of fluorescence intensity confirmed that TGF-β and leptin stimulation induced fibrosis, with significantly increased vimentin expression and decreased E-cadherin expression (Figures 5C, D), validating the models. Lunasin treatments did not alter EMT markers expression or cell migration (Figures 4, 5), suggesting it didn't directly interfere fibrosis in A549 cell. However, lunasin reduced TGF-β production (Figure 2), speculating a potential effect at the early stage of fibrosis in A549 cells. Additional analyses such as histological assessments, Masson's trichrome staining, or α-SMA and collagen I staining will be needed in future studies substantiate this conclusion.

### 3.5 Lunasin inhibited pro-inflammatory and profibrotic cytokines in the lungs of HF-fed mice

**Figure 6:**
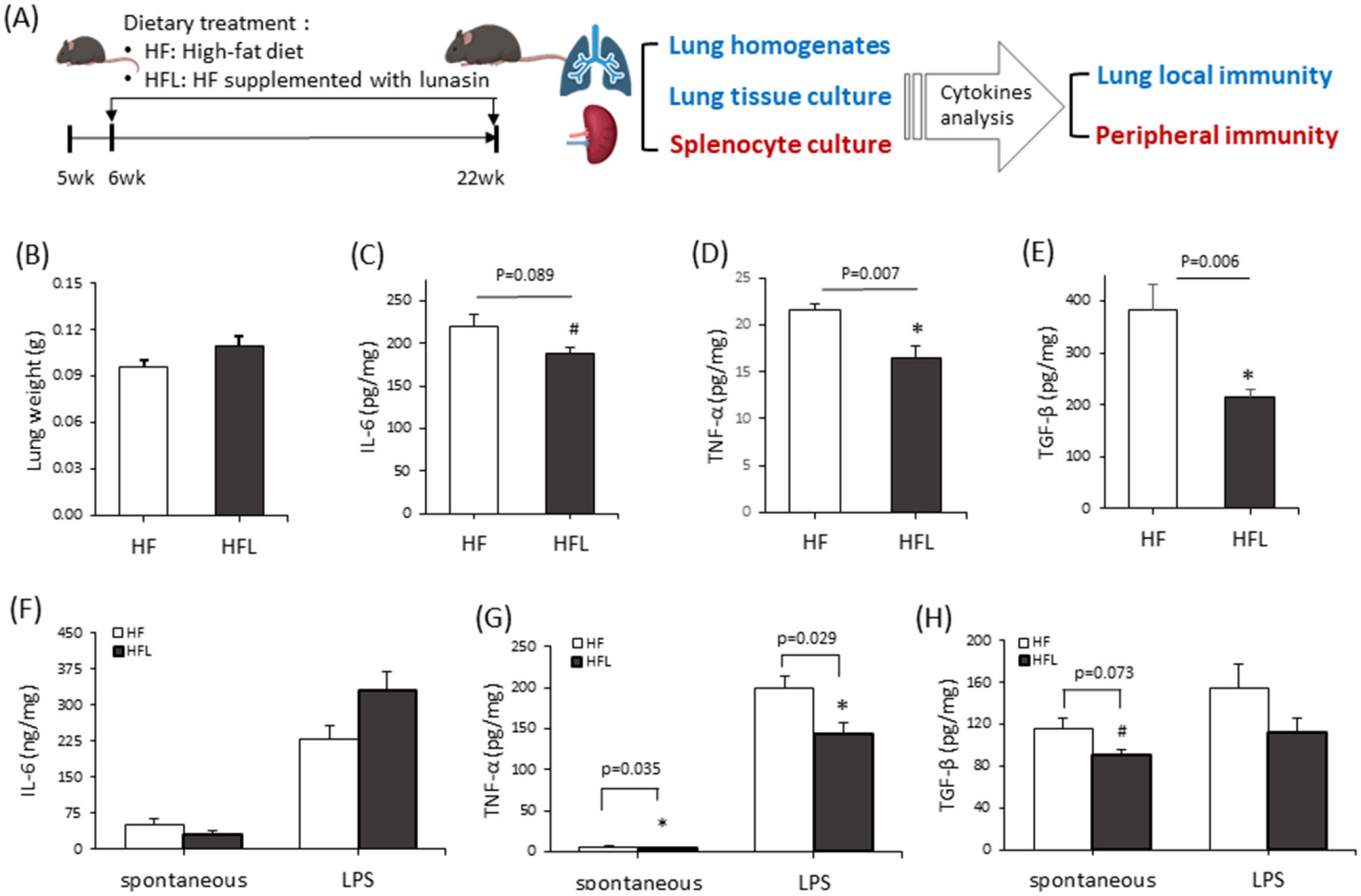
Lunasin regulates inflammatory cytokine secretion in the lungs of mice fed with the high-fat diet. C57BL/6 mice were fed the high-fat diet supplemented with lunasin until they were 22 weeks old. **(A)** The design and process of the animal experiment. **(B)** The lung weights of mice. The levels of cytokines **(C)** IL-6, **(D)** TNF-α, and **(E)** TGF-β in the supernatant of the lung tissue homogenate were analyzed via ELISA. In an *ex vivo* study, the levels of cytokines **(F)** IL-6, **(G)** TNF-α, and **(H)** TGF-β in the culture media of lung tissues were analyzed via ELISA. Cytokine secretion was normalized based on individual protein concentration in each tissue. Data are presented as the mean ± SEM of five to six mice from each group. Statistical analysis was based on Student's *t-*tests. *P < 0.05 vs. HF group; ^#^*P* < 0.1 vs. HF group. HF, high-fat diet; HFL, HF supplemented with lunasin.

To evaluate whether the anti-inflammatory effects of lunasin observed *in vitro* can be reproduced *in vivo*, C57BL/6 mice were fed with an HF diet supplemented with a lunasin-enriched soy protein isolate. Lung and spleen tissues were collected to assess local and systemic immune responses. Lung tissues were used to assess the inflammation in the targeted area, while spleens showed the immune responses around the whole body (Figure 6A). At the end of the study, the lung weight (Figure 6B) and body weight of mice (Supplementary material 2) did not differ among groups, indicating that lunasin does not affect lung mass or weight gain in mice. However, lunasin ameliorated adipocyte size and hepatic steatosis, as indicated by H&E staining and quantitative analysis in mice fed a high-fat diet (Supplementary material 2), suggesting that lunasin modified obesity-related pathological changes. Cytokine IL-6, TNF-α, and TGF-β levels in lung homogenates from mice were analyzed via ELISA. Mice fed with the HF diet with lunasin supplementation showed significantly lower TNF-α and TGF-β levels (*P* < 0.05) and mildly influenced IL-6 (*P* = 0.089) compared with the HF group (Figures 6C–E). Consistently, in *ex vivo* lung culture supernatants from lung tissue, lunasin supplementation significantly reduced LPS-induced TNF-α production (*P* < 0.05) and slightly reduced TGF-β levels (*P* = 0.073; Figures 6G, H) in the HFL group compared with the HF group, while IL-6 was unchanged (Figure 6F). The level of MCP-1 in lung tissue was not affected by lunasin treatment (data not shown). Together, these results demonstrate that lunasin supplementation attenuates pulmonary inflammation by suppressing the pro-inflammatory cytokine TNF-α and the profibrotic mediator TGF-β in mice fed high fat and high fructose diet.

### 3.6 Lunasin regulated cytokine secretion in the spleens of HF-fed mice

**Figure 7:**
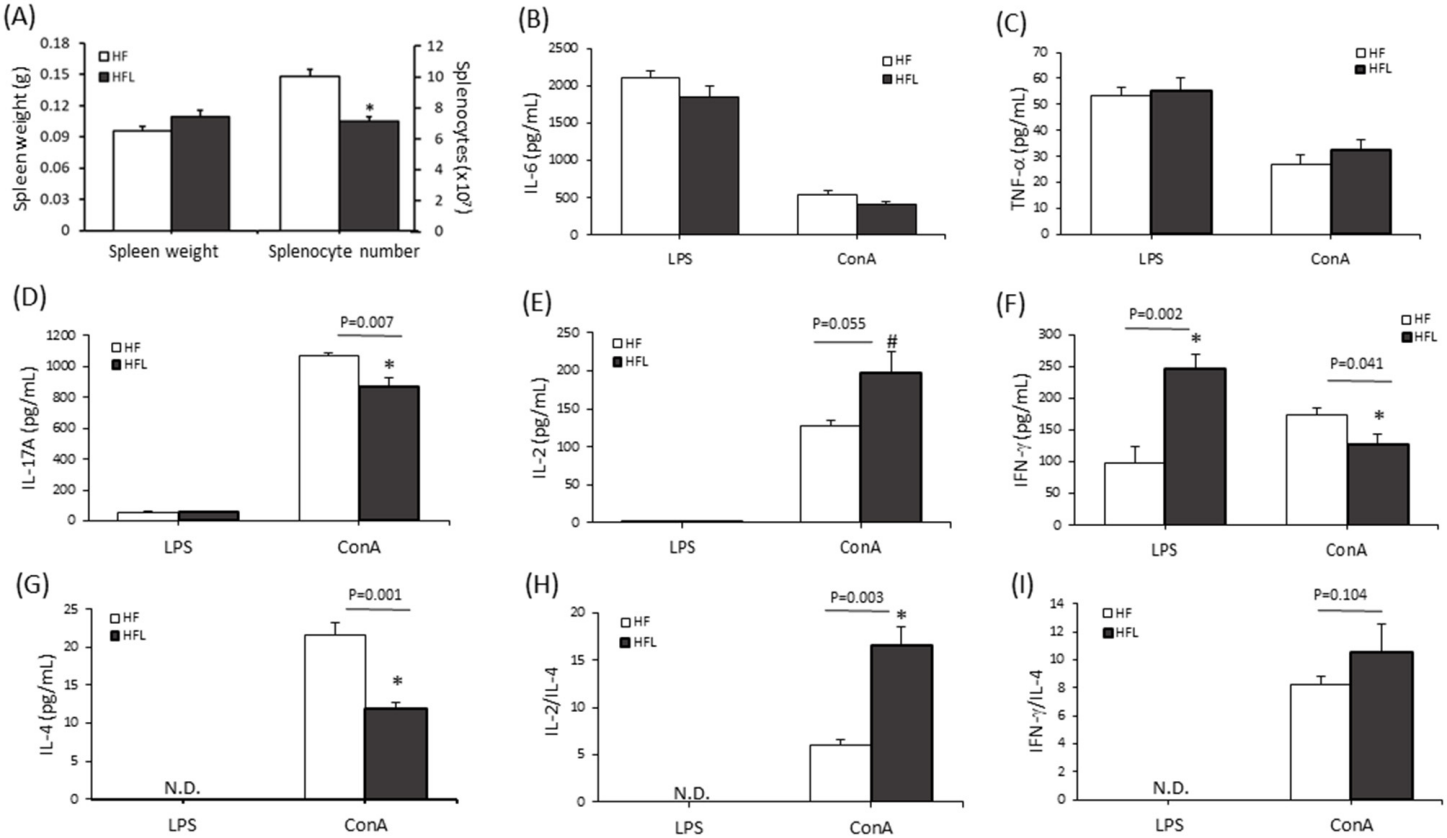
Lunasin regulated cytokines secretion in splenocytes of mice fed with the high-fat diet. C57BL/6 mice were fed with the high-fat diet supplemented with lunasin until they were 22 weeks old. **(A)** The spleen weight and the number of splenocytes in mice. The levels of cytokines **(B)** IL-6, **(C)** TNF-α, **(D)** IL-17A, **(E)** IL-2, **(F)** IFN-γ, and **(G)** IL-4 in the supernatants of cultured splenocytes were analyzed via ELISA. The ratio of Th1/Th2 was calculated as **(H)** IL-2/IL-4 and **(I)** IFN-γ/IL-4. Data are presented as the mean ± SEM of six mice in each group. Statistical analysis was based on Student's *t*-tests. *P < 0.05 vs. HF group; ^#^*P* < 0.1 vs. HF group. HF, high-fat, high-fructose diet; HFL, HF supplemented with lunasin.

The immunomodulatory effects of lunasin were assessed by measuring the secretion of cytokines IL-6, TNF-α, IL-17A, IL-2, IFN-γ, and IL-4 in the supernatants of cultured splenocytes, i.e., the mitogens LPS-stimulated B lymphocytes and ConA-stimulated T lymphocytes. Both mitogens markedly increased cytokine secretion (Figure 7). The splenic weights of the mice were not different between the two groups. However, splenocyte numbers in the HFL group were lower than those in the HF group (*P* < 0.05; Figure 7A). In ConA-stimulated splenocytes in the HFL group, the production of IL-17A, IFN- γ, and IL-4 was decreased (*P* < 0.05), but the IL-2 level was slightly elevated compared to that in the HF group (*P* = 0.055; Figures 7B–G). In contrast, mice in the HFL group showed significantly higher IFN-γ secretion in LPS-stimulated splenocytes, while this level was lower in ConA-stimulated splenocytes. Interestingly, the HFL group exhibited increased levels of T helper (Th)1 cytokine IL-2 and reduced levels of Th2 cytokine IL-4. In order to determine the balance of Th1/Th2 cells, the ratio of IL-2/IL-4 and IFN-γ/IL-4 was calculated to confirm that lunasin supplementation significantly enhanced Th1 polarization (Figures 7H, I). In summary, lunasin regulated systemic immunity by promoting Th1 responses and suppressing pro-inflammatory IL-17A level in the spleens of mice fed with an HF diet, suggesting to ameliorate obesity-related chronic inflammation and immune decline.

## 4 Discussion

**Figure 8:**
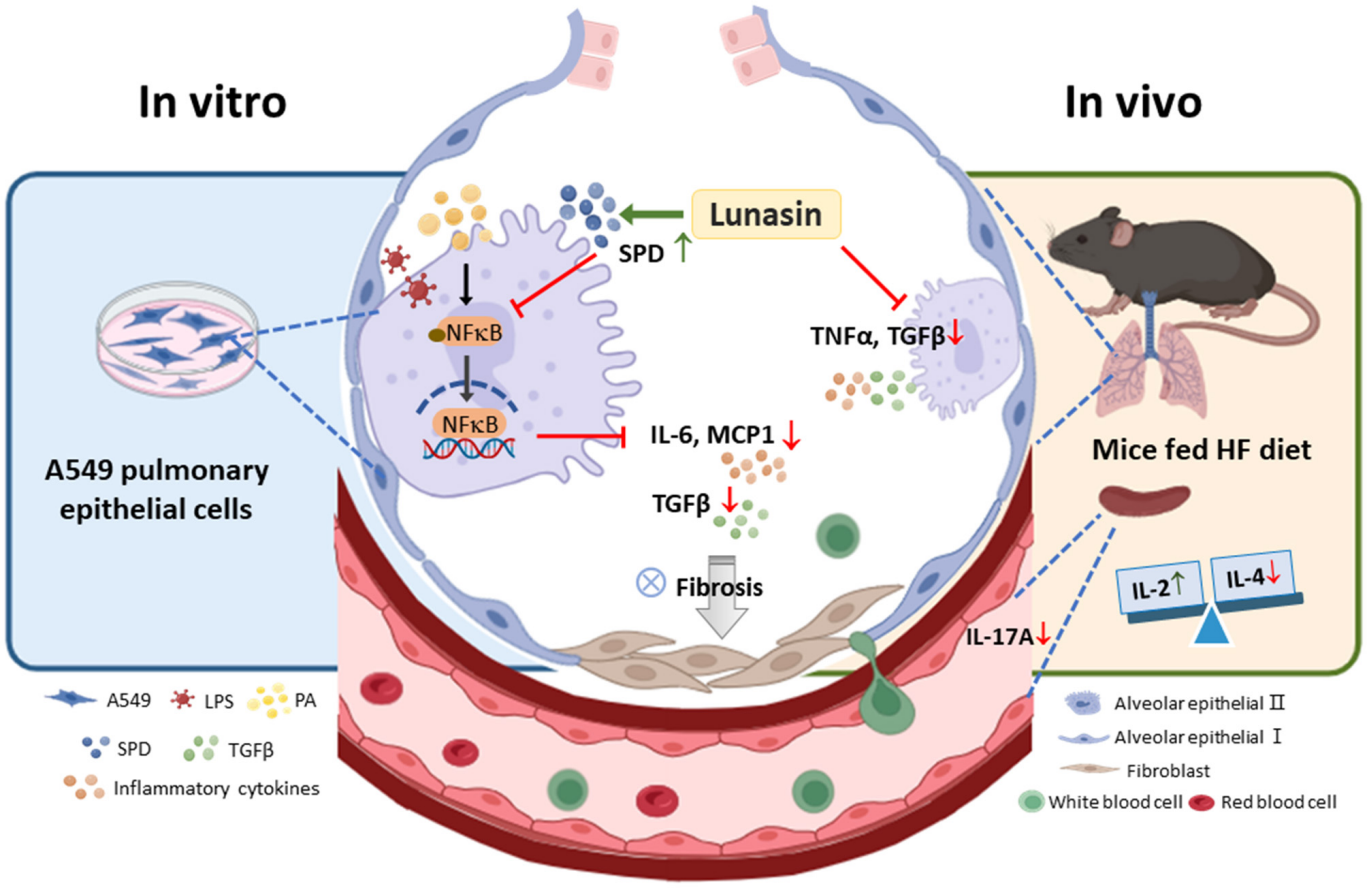
Lunasin alleviates pulmonary inflammation in A549 alveolar epithelial cells and mice in obese-mimicking conditions. Lunasin reduced inflammatory IL-6, MCP-1, and profibrotic TGF-β cytokines through enhancing SP-D expression and inhibiting NF-κB signaling in A549 cells. In mice fed the HF diet, lunasin inhibited the production of pulmonary cytokines IL-6, TNF-α, and TGF-β, improved splenic immune balance, and reduced IL-17A levels. This highlights the potential application of lunasin as a functional food compound in mitigating pulmonary inflammation associated with obesity.

Obesity is a chronic disease that negatively impacts health by impairing the function of multiple organs, including the lungs. Dysfunctional adipose tissue contributes to pulmonary pathology, making it essential to explore the potential of bioactive compounds that can modulate lung inflammation and fibrosis in obesity-related models. In this study, *in vitro*, lunasin suppressed IL-6, MCP-1, and TGF-β production in A549 cells by upregulating SP-D and inhibiting NF-κB signaling, although it did not affect the characteristics of fibrosis. *In vivo*, mice fed with an HF diet enriched with lunasin showed reduced pulmonary cytokine TNF-α and TGF-β levels, accompanied by an enhanced Th1 immune response and reduced IL-17A secretion in splenocytes. These findings reveal, for the first time, that lunasin plays a pivotal role in reducing inflammatory and profibrotic cytokines production in pulmonary epithelial cells and lung tissues in the HF-fed mice, suggesting its potential in preventing obesity-associated respiratory complications. A proposed mechanism illustrates how lunasin affects pulmonary inflammation and fibrosis in obesity-related models (Figure 8).

Alveolar type II (ATII) cells play a critical role in the structural maintenance, defense, and repair of the lungs, performing essential functions such as epithelial regeneration, surfactant secretion, and immune regulation. These cells are involved in protection against pathogen exposure and defenses against respiratory diseases (29). A549 cells, which exhibit ATII-like characteristics after long-term culture (30), were used in this study to model the pulmonary alveolus due to the relevance of ATII dysfunction in lung diseases.

Adiposity triggers chronic low-grade inflammation by releasing adipokines, cytokines, and chemicals such as TNF-α, IL-6, CRP, leptin, and MCP-1, while reducing the level of anti-inflammatory adiponectin (4). Accordingly, these mediators recruit immune cells, such as macrophages, B cells, and T cells, which infiltrate adipose tissues on a large scale and participate in the development of several diseases, such as metabolic and respiratory diseases (3, 31). In this study, high-glucose media, PA, and LPS were used to mimic obese conditions *in vitro*. A549 cells cultured in high-glucose media showed rising IL-6 and MCP-1 levels. Lunasin inhibited IL-6 and MCP-1 secretions in normal-glucose media via PA or LPS stimulation, particularly by reducing TGF-β levels in high-glucose media with LPS, indicating that lunasin effectively reduces pro-inflammatory and pro-fibrotic mediators in A549 cells under obesity-related conditions.

Research has demonstrated that lunasin exhibits anti-inflammatory effects across various models. The first study on this topic reported that lunasin reduced inflammatory mediators in RAW 264.7 macrophages (32), and similar effects were reported in HepG2, Caco-2, adipocytes, breast cancer cells, and C2C12 myotubes (18, 33–36). Lunasin also inhibits cytokine crosstalk between macrophages and adipocytes (37), suppresses ROS and MCP-1 production in LPS stimulation, and boosts the immune responses of macrophages in obese conditions (19). *In vivo*, lunasin has been shown to reduce inflammatory bowel disease in mice by suppressing caspase-1, IL-1 β, and IL-18 secretion, and possibly through modulation of the NLRP3 inflammasome (38), as well as in those with diet-induced obesity (39). Consistent with prior reports in macrophages, hepatocytes, and adipocytes, as well as in mouse models of inflammatory diseases, these findings reinforce lunasin's broad anti-inflammatory activity and support its potential as a therapeutic agent against obesity-related pulmonary inflammation.

Surfactant proteins are essential in maintaining lung homeostasis and immune defense. SP-A and SP-D primarily modulate the innate immune responses, implicating in neutralizing pathogens, and drive pro- or anti-inflammatory signaling (40). SP-D recognizes exposed highly conserved glycosidic residues on pathogens and triggers their clearance through agglutination, receptor blocking, and opsonization (41). A laboratory study demonstrated that prolonged exposure to organic barn dusts caused chronic inflammation and increased GRP116 receptor expression, subsequently reducing SP-D levels in mouse lungs and confirming the mechanism in A549 cells (41). However, increased SP-D has been observed in COVID-19 patients (42) and in mice with acute lung injury (43), suggesting its potential as a biomarker for pulmonary inflammation and damage.

Based on the effects observed, the mechanism behind lunasin's action may involve the regulation of the NF-κB signaling pathway. SP-D-knockout mice infected with Pseudomonas aeruginosa exhibited exacerbated lung and kidney injury and inflammation due to active NF-κB signaling, compared with wild-type mice (44). Similarly, recombinant human SP-D has been shown to bind the TLR4/MD-2 complex in chondrocytes, thereby suppressing TLR4-mediated NF-κB activation and reducing LPS-induced inflammation (45). Accordingly, SP-D suppresses inflammatory mediators by attenuating NF-κB signaling in response to pathogen infection or injurious challenges. Consistently with this, our study demonstrated that LPS reduced SP-D expression in A549 cells, whereas lunasin restored SP-D levels by inhibiting NF-κB phosphorylation and subsequent pro-inflammatory cytokine production. Although lunasin inhibited IL-6 secretion in PA stimulation, but it showed no effect under LPS challenge in A549 cells. The limitations of this experiment refer to the individual *in vitro* model and the possibility of each stimulator having a different effective dosage, duration, and signaling pathway. Moreover, lunasin has also been demonstrated to have anti-inflammatory properties through NF-κB signaling suppression in macrophages (17, 25, 46).

Pulmonary fibrosis is closely linked to chronic inflammation, and obesity-related factors may exacerbate this process through inflammatory mediators such as TNF-α, TGF-β, and MCP-1. Inflamed lungs promote these mediators, driving cell types toward profibrotic transitions (4, 6). Natural products can slow the progression of pulmonary fibrosis by reducing TGF-β production and collagen deposition, providing alternative therapeutic choices with fewer side effects (47). In this study, TGF-β and leptin significantly enhanced cell migration and vimentin expression, while reducing E-cadherin, indicating the occurrence of EMT. Although lunasin treatment reduced TGF-β production, it did not directly affect EMT marker expression and cell migration, suggesting that its anti-fibrotic effects may act primarily at the early stage of profibrotic mediator induction rather than on downstream EMT processes.

Dietary factors can modulate systemic and pulmonary inflammation, thereby potentially attenuating fibrosis in obesity (11). To validate lunasin's effects *in vivo*, mice fed with an HF diet were examined with regard to immune responses in their lung and spleen tissues. Lunasin supplementation reduced levels of TNF-α and TGF-β in the lungs, consistent with the *in vitro* findings. The impact of lunasin was also observed in the spleen, which is the most significant organ in peripheral immunity, performing a broad range of immunological functions for the host (48). Obesity-induced adipose tissue inflammation is partly characterized by an imbalance in Th and regulatory T (Treg) cells (31). In this study, lunasin increased Th1 cytokines (IL-2, IFN-γ), elevated the Th1/Th2 ratio, and suppressed IL-17A, suggesting that lunasin restores immune balance in HF-fed mice by promoting Th1 responses and dampening pro-inflammatory signaling. Additionally, the composition of gut microbes is linked to intestinal permeability and barrier integrity, which, in turn, can affect pulmonary health. As a result, we are going to conduct a study to investigate the effects of lunasin-enriched soy protein isolate on the gut microbiome and barrier function in mice that are fed a high-fat diet to have a comprehensive mechanistic understanding.

IL-2 is a critical growth factor for T-cell proliferation and immune homeostasis, making raising important to restore immune function in obesity (49). As noted in our previous study, the intraperitoneal administration of lunasin in obese mice reduces MCP-1, IL-1β, and TNF-α levels in peritoneal macrophages, while enhancing IL-2 and IFN-γ secretion and reducing splenocyte numbers (39). These results are consistent with this study, where dietary supplementation with a lunasin-enriched soy protein isolate similarly promoted IL-2 and IFN-γ production. Supporting evidence from clinical and immunological studies further highlights lunasin's immunomodulatory potential. In lymphoma patients, lunasin enhanced granzyme B and INF-γ production in activated natural killer cells (50) and promoted the maturation of dendritic cells as a vaccine adjuvant (51).

Obesity impairs immune responses to pathogenic infections (52) and is an established risk factor for poor outcomes in COVID-19. Reduced production of antiviral cytokines, including IFN-α, IFN-γ, and TNF-α, has been observed in lung epithelial, and immune cells from obese hosts (53). However, IFN- γ secreted by T cells in obese mice is associated with enhanced insulin resistance and hepatic steatosis (54). In this study, lunasin supplementation differentially regulated IFN-γ production, enhancing it in response to LPS stimulation but reducing it under ConA activation, suggesting that lunasin plays a context-dependent immunomodulatory role. This indicates that lunasin may strengthen host defenses against infection while mitigating T cell-driven metabolic inflammation in obesity.

IL-2 and IFN-γ are characteristic Th1 cytokines, and IL-4 and IL-5 are hallmark Th2 responses. The production ratio of IL-2/IL-4 and IFN-γ/IL-4 is commonly used to assess Th1/Th2 balance, with higher ratios indicating Th1 dominance and lower ratios reflecting a Th2 shift. Several studies have applied these ratios as reliable indicators of immune status. The IL-2/IL-4 ratio in EL-4 T cells has been used to evaluate Th1/Th2 balance in an obese model (33), while the IFN-γ/IL-4 ratio provides a more accurate reflection of Th1/Th2 immune responses than single cytokine measurements (55).

Another cytokine, IL-17A, is primarily secreted by Th17 cells and innate lymphoid cells, is elevated in obesity, and promotes systemic inflammation, insulin resistance, and metabolic dysfunction (56). It also acts directly on the airway smooth muscle and respiratory epithelium, contributing to bronchial hyper-reactivity and epithelial injury, thereby worsening outcomes in obesity and COVID-19 (57). Research has demonstrated that the neutralization of IL-17A could represent a potential therapeutic strategy to reduce inflammation (58). In this study, lunasin reduced IL-17A secretion while enhancing Th1 cytokine production, effectively shifting the Th1/Th2/Th17 balance toward improved immune function and reduced pulmonary inflammation. Based on these data, lunasin improves immune balance in obesity by strengthening Th1 responses, restraining excessive IL-17A activity, and supporting T cell-mediated homeostasis in mice fed high fat/fructose diet.

## 5 Conclusions

Obesity is accompanied by chronic low-grade inflammation, linked to pulmonary inflammation and fibrosis, as well as various respiratory diseases (1, 2, 4). Lunasin, a seed-derived peptide, exhibits several biofunctions (15–17). This study reveals for the first time that lunasin inhibits the secretion of the pro-inflammatory cytokines IL-6, MCP-1, and TNF-α and the profibrotic mediator TGF-β in pulmonary epithelial cells through an increase in SP-D and a decrease in NF-κB signaling. In mice fed the high-fat diet, lunasin supplementation reduced pulmonary inflammatory cytokines production and promoted Th1 immune responses and reduced IL-17A secretion in splenocytes, suggesting improved immune defense and balance. The limitations of the *in vitro* study are that PA and LPS may activate different pathways and have varying durations and effects. Moreover, the small sample size and biological variance are factors to consider *in vivo*. The advanced molecular mechanisms underlying its effects could be investigated in the future, such as testing lunasin with SP-D or NF-κB inhibitors. Additionally, gut microbial composition is associated with intestinal permeability and barrier integrity, further influencing pulmonary health. Therefore, the gut-lung axis represents a plausible mechanism for future investigation. Based on these results, lunasin has showed potential anti-inflammatory and immunomodulating properties. In summary, this study emphasizes lunasin as a promising functional food component for addressing obesity-related pulmonary inflammation.

## Data Availability

The datasets presented in this study can be found in online repositories. The names of the repository/repositories and accession number(s) can be found in the article/Supplementary material.
