# Supplementary material for: Lunasin alleviates pulmonary inflammation in A549 alveolar epithelial cells and C57BL6/J mice in obese-mimicking conditions
Source: Front Nutr. 2026 Feb 5;13:1732250. doi: 10.3389/fnut.2026.1732250 (PMC12916388; doi:10.3389/fnut.2026.1732250)
Supplement: Supplementary file 1 [file Supplementary_file_1.pdf]

Supplementary 1.

The dietary composition of the High-fat diet and HF diet supplemented with lunasin-enriched soy protein isolated.

Ingredient	High-fat diet	HF diet supplemented with lunasin
Calorie (kcal/kg)	4724	4724
	Calorie (%) of diet	
Carbohydrate	35.2	35.2
Protein	19.8	19.8
Fat	45.0	45.0
	Weight (g/kg) of diet	
Cellulose	57	57
Sucrose	100	100
Fructose	316	316
Casein	230	221
L-cystine	4	4
Soybean oil	10	10
Lard	226	226
AIN-93G mineral mix	42	42
AIN-93G vitamin mix	12	12
Choline bitartrate	3	3
Lunasin-enriched soy protein (LES)[1]	-	15.4

[1] LES was purified from PRO-FAM®974 Isolated Soy Protein (ADM, Chicago, USA).
LES contains 3.1% of lunasin that can provide 483 mg lunasin per kg of diet.
