# Supplementary material for: Lunasin alleviates pulmonary inflammation in A549 alveolar epithelial cells and C57BL6/J mice in obese-mimicking conditions
Source: Front Nutr. 2026 Feb 5;13:1732250. doi: 10.3389/fnut.2026.1732250 (PMC12916388; doi:10.3389/fnut.2026.1732250)
Supplement: Supplementary file 2 [file Supplementary_file_2.pdf]

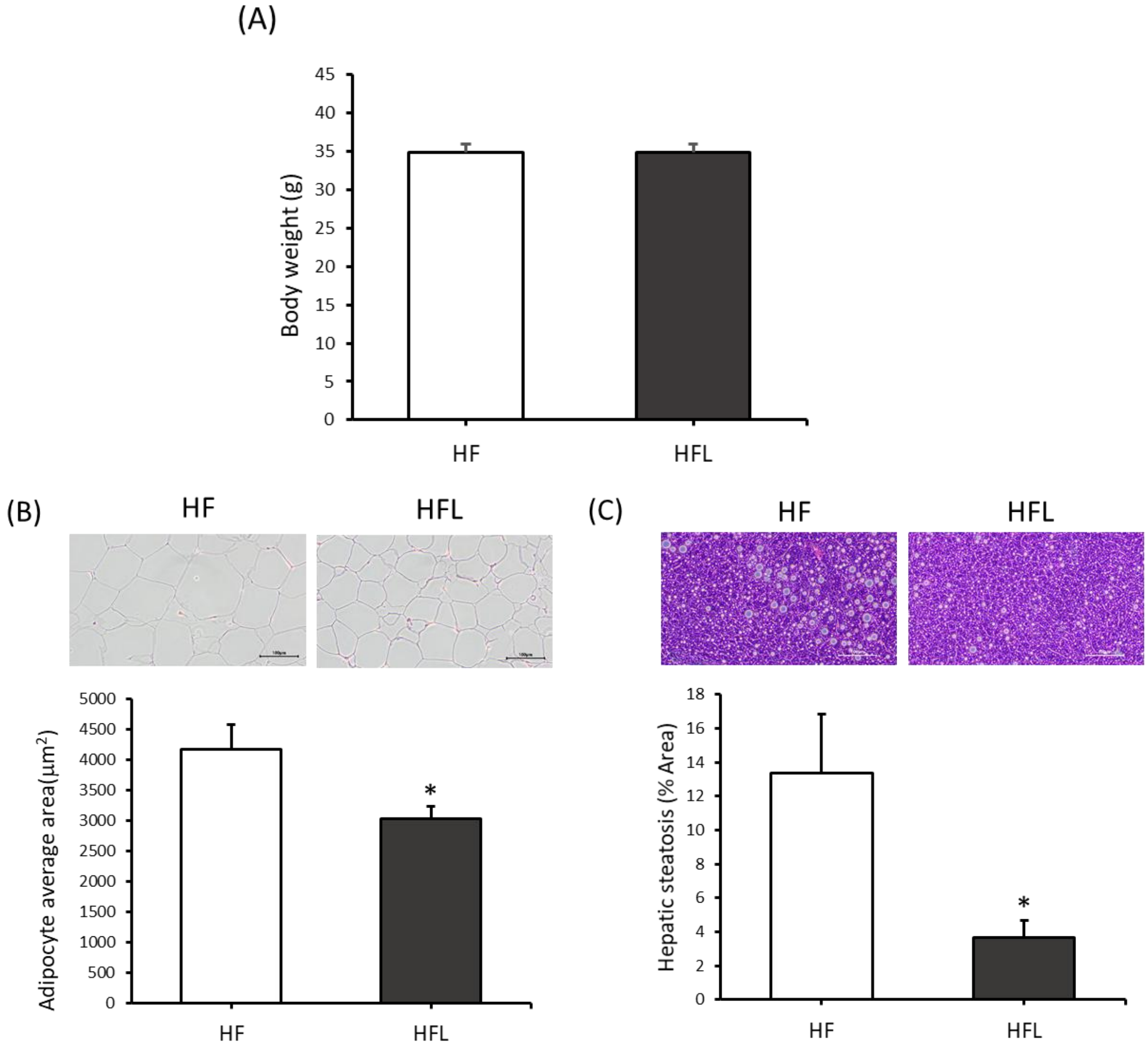

Supplementary 2. Lunasin ameliorates adipocytes size and hepatic steatosis in mice fed the high-fat diet. C57BL/6 mice were fed with high-fat diet supplemented with lunasin until 22-weeks-old. (A) The total body weight of mice. (B) Photographs of epididymal white adipose tissue section by H&E staining and quantitative analysis. (C) Photographs of liver section by H&E staining and quantitative analysis. Data are presented as the mean ± SEM from six mice in each group. Statistical analysis was based on a Student's t-test. * $P < 0.05$ vs. HF group. HF, high-fat diet; HFL, HF supplemented with lunasin. Scale bar: 100 μm.
