# Supplementary material for: Lunasin alleviates pulmonary inflammation in A549 alveolar epithelial cells and C57BL6/J mice in obese-mimicking conditions
Source: Front Nutr. 2026 Feb 5;13:1732250. doi: 10.3389/fnut.2026.1732250 (PMC12916388; doi:10.3389/fnut.2026.1732250)

Supplementary 3. All data used for this study was available in this file.

Lunasin alleviates pulmonary inflammation in A549 alveolar epithelial cells and C57BL6/J mice in obese-mimicking conditions.

Wan-Sheng Chang, Pei-Ying Huang, Chia-Chien Hsieh*
Department of Biochemical Science & Technology, College of Life Science, National Taiwan University, TW

(A)

**Cell Viability**	fold of control							
**Lunasin**	1	2	3	4	**mean**	**SD**	**SEM**	**P-value**
0	100	100	100	100	100	0.00	0.00	
1	115	94	95	103	102	9.59	4.80	0.739
5	111	94	98	95	100	7.88	3.94	0.936
10	102	96	82	104	96	9.78	4.89	0.455
25	98	88	93	96	94	4.47	2.23	0.064
50	76	84	73	98	83	11.02	5.51	0.051
100	85	51	74	103	78	21.97	10.98	0.143
200	77	58	65	78	69	9.79	4.89	0.008

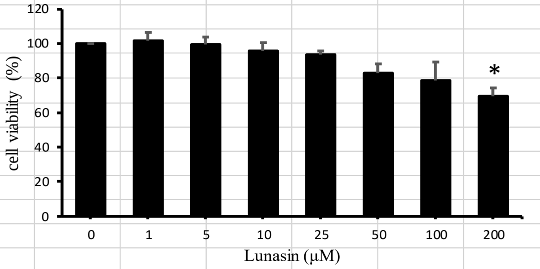

(B)

cell ciability	fold of control							
PA	1	2	3	4	mean	SD	SEM	P-value
0	1.0	1.0	1.0	1.0	1.00	0.0	0.00	
50	1.0	1.1	1.1	1.0	1.06	0.0	0.02	0.134
100	1.5	0.9	1.1	0.9	1.07	0.3	0.14	0.53
200	0.8	0.8	0.9	0.8	0.80	0.0	0.02	0.006
500	0.6	0.6	0.4	0.4	0.48	0.1	0.05	0.003
1000	0.1	0.1	0.1	0.1	0.07	0.0	0.01	0.0001

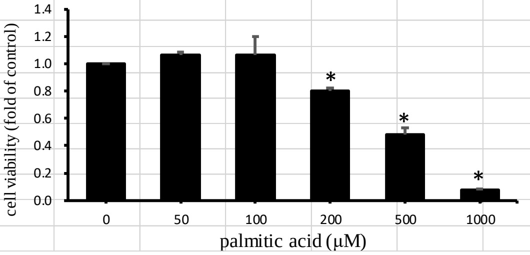

(C)

**Cell Viability**		fold of control							
**treatment**	**μg/ml**	**1**	**2**	**3**	**4**	**mean**	**SD**	**SEM**	**P-value**
control	0	100	100	100	100	100	0.00	0.00	
LPS	0.1	116	93	99	108	104	10.28	5.14	0.191
LPS	0.5	108	105	88	103	101	9.19	4.60	0.513
LPS	1	111	100	99	111	105	6.93	3.47	0.056
LPS	2	104	106	87	100	99	8.73	4.36	0.633
LPS	5	108	104	92	99	101	6.95	3.48	0.49
LPS	10	102	101	84	98	96	8.60	4.30	0.735
LPS	20	108	88	80	95	93	11.80	5.90	0.332

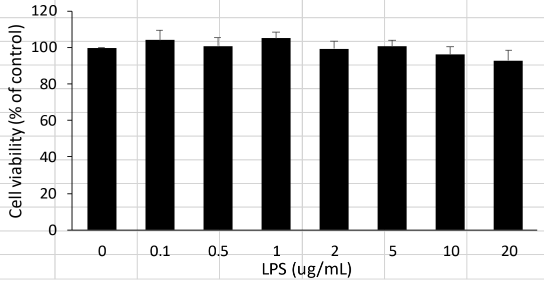

(D)

**IL-6 final concentration (pg/ml)**								
**Lunasin**	**1**	**2**	**3**	**4**	**mean**	**SD**	**SEM**	**P-VALUE**
0	61.92	63.83	49.06	38.59	53	11.83	5.91	
1	50.49	44.30	36.68	50.49	45	6.56	3.28	0.301
5	43.35	38.11	46.68	52.87	45	6.18	3.09	0.271
10	54.78	34.30	41.44	50.97	45	9.27	4.63	0.329
25	38.11	32.40	47.16	46.68	41	7.13	3.57	0.126
50	26.68	33.83	41.44	40.49	36	6.85	3.42	0.041
100	33.83	65.73	55.25	54.30	52	13.35	6.67	0.908
200	65.73	60.49	53.35	52.87	58	6.16	3.08	0.502

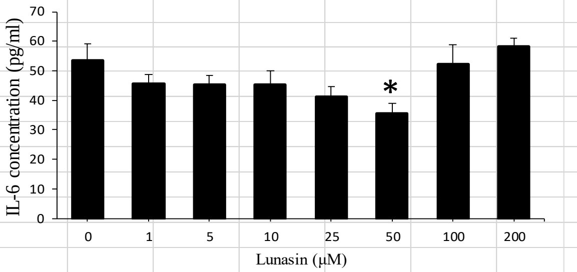

(E)

IL-6	IL-6 concentration (pg/ml)						
PA (uM)	1	2	3	mean	SD	SEM	P-value
0	23.87	0.06	15.78	13.24	12.1	6.99	
50	23.40	18.16	27.68	23.08	4.8	2.75	0.260
100	31.97	27.21	37.21	32.13	5.0	2.89	0.067
200	33.40	29.59	41.49	34.83	6.1	3.51	0.051
500	36.73	44.83	68.63	50.06	16.6	9.58	0.036
1000	40.06	57.68	84.83	60.86	22.5	13.02	0.032

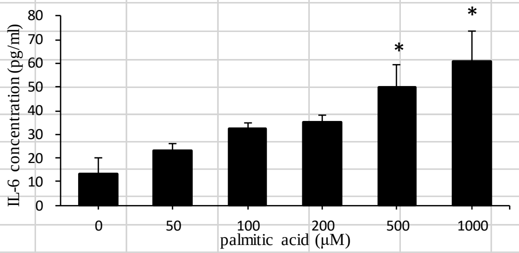

(F)

**IL-6 concentration (pg/ml)**								
**LPS μg/ml**	**1**	**2**	**3**	**4**	**mean**	**SD**	**SEM**	P-value
**0**	21.49	23.87	0.00	0.00	11	13.13	6.57	
**0.1**	16.25	16.73	9.11	17.68	15	3.93	1.97	0.618
**0.5**	24.35	42.92	19.59	31.97	30	10.18	5.09	0.069
**1**		42.44	53.40	44.83	47	5.76	3.33	0.008
**2**	31.02	75.30	51.97	41.49	50	18.95	9.47	0.015
**5**	52.92	61.49	65.30	59.59	60	5.18	2.59	0.000
**10**	64.83	63.87	75.30	87.52	73	11.05	5.53	0.000
**20**	79.59	92.44	98.16	85.30	89	8.12	4.06	0.000

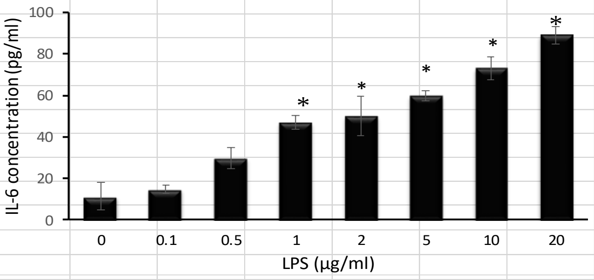

Figure 2.

(A)

高低糖比(對spontaneous的零點)

IL-6		1	2	3	4	mean	SD	SEM	model p-value	LG/HG p-value
spontaneous	0	1.00	1.00	1.00	1.00	1.00	0.00	0.00		
	HG	0.48	1.11	1.76	0.95	1.07	0.53	0.27	0.79	
PA	low Glu	3.42	1.83	2.79	3.11	2.79	0.69	0.34	0.002	
	high Glu	1.70	2.28	2.16	2.18	2.08	0.26	0.13	0.004	0.102
LPS	low Glu	22.36	10.80	15.22	11.82	15.05	5.23	2.61	0.013	
	high Glu	7.89	11.74	13.47	16.66	12.44	3.65	1.83	0.008	0.445
TGFβ	low Glu	1.91	1.99	5.57	6.04	3.88	2.24	1.12	0.082	
	high Glu	9.00	13.35	22.01	11.12	13.87	5.71	2.86	0.02	0.017

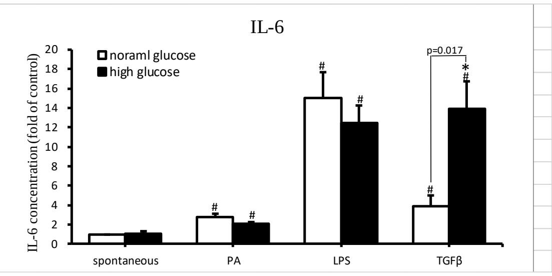

(B)

高低糖比(對spontaneous的零點)

MCP-1		1	2	3	4	mean	SD	SEM	model p-value	LG/HG p-value
spontaneous	0	1.00	1.00	1.00	1.00	1.00	0.00	0.00		
	HG	1.12	1.41	1.38	1.40	1.33	0.14	0.07	0.018	
PA	low Glu	1.34	1.21	0.75	1.14	1.11	0.25	0.13	0.412	
	high Glu	1.44	1.65	1.27	1.28	1.41	0.18	0.09	0.02	0.102
LPS	low Glu	2.13	2.03	2.45	2.24	2.22	0.18	0.09	0.001	
	high Glu	2.88	3.92	3.82	3.74	3.59	0.48	0.24	0.002	0.002
TGFβ	low Glu	0.96	1.46	1.34	0.99	1.19	0.25	0.13	0.234	
	high Glu	1.07	1.27	1.44	1.39	1.29	0.16	0.08	0.037	0.504

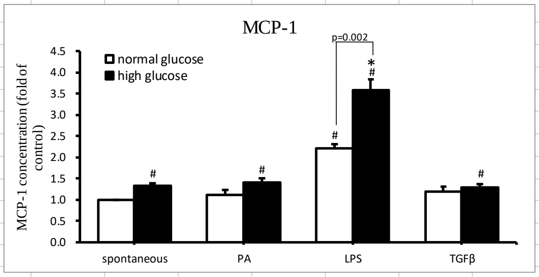

(C)

高低糖比(對spontaneous的零點)

TGF-β		1	2	3	4	mean	SD	SEM	model p-value	LG/HG p-value
spontaneous	0	1.00	1.00	1.00	1.00	1.00	0.00	0.00		
	HG	0.56	0.82	0.92	1.07	0.84	0.22	0.11	0.239	
PA	low Glu	1.09	1.00	1.68	1.68	1.36	0.37	0.18	0.141	
	high Glu	0.82	0.80	1.48	1.66	1.19	0.45	0.22	0.454	0.571
LPS	low Glu	1.54	1.66	2.74	1.65	1.90	0.56	0.28	0.05	
	high Glu	1.23	1.48	1.50	2.44	1.67	0.53	0.27	0.087	0.574
TGFβ	low Glu	5.80	5.57	8.42	5.45	6.31	1.41	0.71	0.005	
	high Glu	5.45	4.79	5.13	4.77	5.03	0.32	0.16	0.000138	0.128

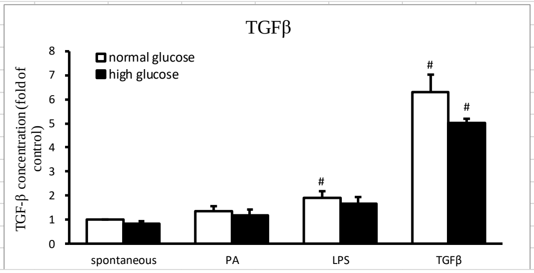

(D)

IL-6		1	2	3	4	mean	SD	SEM	model p-value	Lunasin p-value
spontaneous	0	1.00	1.00	1.00	1.00	1.00	0.00	0.00		
mannitol	+28mM	1.02	1.04	1.39	0.61	1.01	0.32	0.16	0.943	
PA	0	3.42	1.83	2.79	3.11	2.79	0.69	0.34	0.002	
	Lun 5	0.88	0.98	2.59	1.27	1.43	0.79	0.39	0.355	0.041
	Lun 50	1.50	2.19	2.76	1.42	1.97	0.63	0.32	0.054	0.129
LPS	0		10.80	15.22	11.82	12.61	2.31	1.34	0.013	
	Lun 5	14.72	14.55	18.85	14.10	15.56	2.21	1.11	0.001	0.148
	Lun 50	9.08	12.61	19.28	17.42	14.60	4.63	2.31	0.01	0.532

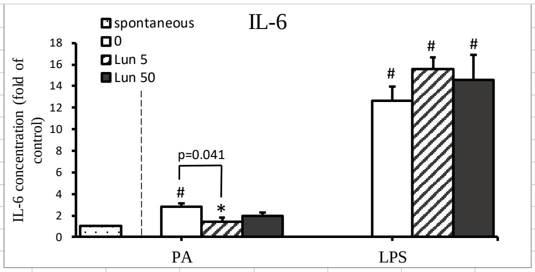

(E)

MCP-1		1	2	3	4	mean	SD	SEM	model p-value	Lunasin p-value
spontaneous	0	1.00	1.00	1.00	1.00	1.00	0.00	0.00		
mannitol	+28mM	1.49	1.63	1.63	1.56	1.58	0.06	0.03	0.000376	
PA	0	1.34	1.21		1.14	1.23	0.10	0.06	0.063	
	Lun 5	0.82	0.87		0.81	0.83	0.03	0.02	0.01	0.003
	Lun 50	0.83	0.88		0.98	0.90	0.07	0.04	0.137	0.011
LPS	0	2.13	2.03	2.45	2.24	2.22	0.18	0.09	0.001	
	Lun 5	1.61	1.86	1.85	1.86	1.80	0.12	0.06	0.001	0.009
	Lun 50	1.53	1.87	2.02	1.85	1.82	0.21	0.10	0.000221	0.027

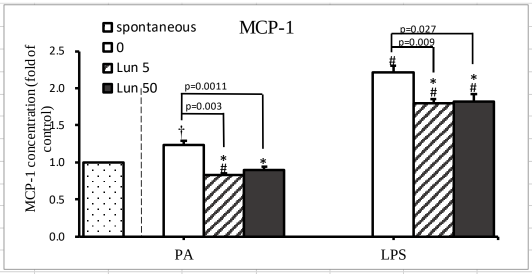

(F)

TGF-β		1	2	3	4	mean	SD	SEM	model p-value	Lunasin p-value
spontaneous	0	1.00	1.00	1.00	1.00	1.00	0.00	0.00		
PA	0	1.47	0.98	1.62	1.55	1.40	0.29	0.14	0.067	
	Lun 5	0.65	1.11	0.07	1.21	0.76	0.52	0.26	0.423	0.073
	Lun 50	2.09	1.01	2.63	1.46	1.80	0.71	0.35	0.111	0.347
LPS	0	2.22	1.81	1.64	2.28	1.99	0.31	0.16	0.008	
	Lun 5	1.62	1.34	1.78	1.28	1.51	0.24	0.12	0.023	0.049
	Lun 50	3.08	1.83	2.25	1.82	2.25	0.59	0.30	0.025	0.47

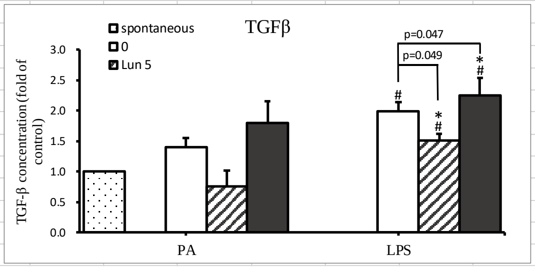

Figure 3.

(B)

SPA

對spontaneous的零點

normal Glu

SP-A		Fold of control						model	lunasin
		1	2	3	mean	SD	SEM	p-value	p-value
spontaneous	0	1.00	1.00	1.00	1.00	0.00	0.00		
	Lun 50	1.54	1.12	0.79	1.15	0.38	0.22	0.52	0.52
PA	0	1.31	1.07	0.84	1.07	0.24	0.14	0.623	
	Lun 50	1.37	1.21	0.68	1.09	0.36	0.21	0.712	0.955
LPS	0	1.23	0.66	0.89	0.93	0.29	0.17	0.677	
	Lun 50	1.52	1.06	0.74	1.10	0.39	0.23	0.67	0.56

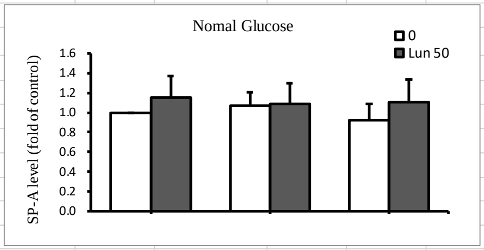

(A)

SPD

對spontaneous的零點

normal Glu

SP-D		Fold of control						model	lunasin
		1	2	3	mean	SD	SEM	p-value	p-value
spontaneous	0	1.00	1.00	1.00	1.00	0.00	0.00		
	Lun 50	0.96	0.82	0.72	0.84	0.12	0.07	0.081	0.081
PA	0	0.71	0.95	1.07	0.91	0.18	0.10	0.475	
	Lun 50	1.16	1.20	0.82	1.06	0.21	0.12	0.664	0.395
LPS	0	0.68	0.67	0.79	0.71	0.06	0.04	0.016	
	Lun 50	1.14	1.06	1.00	1.07	0.07	0.04	0.188	0.003

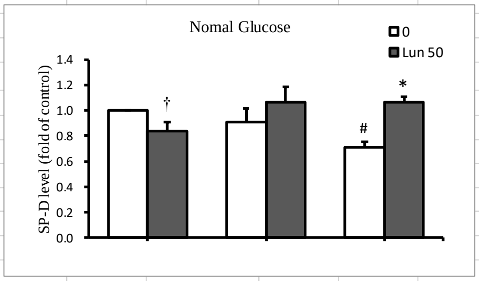

(C,D)

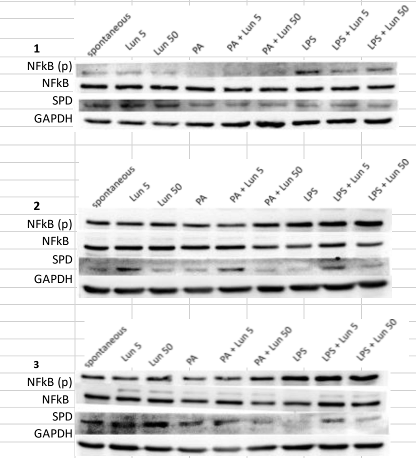

(E, F)

SPD /GAPDH ratio

SPD /GAPDH		1	2	3	mean	SD	SEM	ND p-value	Lunasin p-value
pontaneou	0	1.00	1.00	1.00	1.00	0.00	0.00		
	Lun5	1.20	2.83	0.85	1.63	1.05	0.53	0.41	
	Lun50	1.23	1.20	1.15	1.20	0.04	0.02	0.01	
PA	0	0.50	1.37	0.88	0.92	0.58	0.29	0.755	
	Lun5	0.44	1.93	1.20	1.19	0.75	0.37	0.678	0.326
	Lun50	0.44	0.97	0.47	0.63	0.30	0.15	0.16	0.992
LPS	0	0.70	0.32	0.40	0.47	0.29	0.14	0.045	
	Lun5	0.83	3.66	0.71	1.73	1.67	0.84	0.527	0.321
	Lun50	0.45	1.39	0.48	0.77	0.53	0.27	0.537	0.414

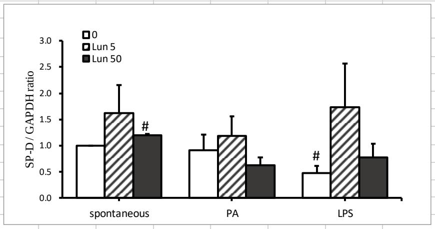

NFkB (p)/NFkB ratio

NFkB (p)/NFkB		1	2	3	mean	SD	SEM	ND p-value	Lunasin p-value
pontaneou	0	1.00	1.00	1.00	1.00	0.00	0.00		
	Lun5	1.45	0.86	1.02	1.11	0.30	0.15	0.269	
	Lun50	1.31	0.94	1.36	1.20	0.23	0.11	0.712	
PA	0	0.81	0.81	0.86	0.83	0.42	0.21	0.897	
	Lun5	0.98	0.66	1.03	0.89	0.20	0.10	0.167	0.288
	Lun50	1.70	1.71	1.88	1.76	0.10	0.05	0.515	0.542
LPS	0	3.17	2.56	2.12	2.62	1.38	0.69	0.006	
	Lun5	1.52	1.87	1.84	1.74	0.19	0.10	0.021	**0.054**
	Lun50	2.53	3.27	2.15	2.65	0.57	0.29	0.007	0.944

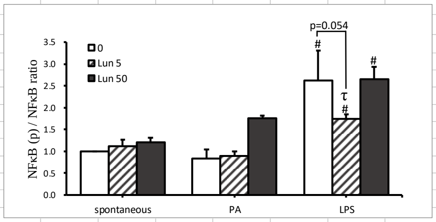

Figure 4.

fibrosis: scratch wound assay

Migration index (%) = [(the initialized width of the scratch) - (the final width of the scratch)]/(the initialized width of the scratch)×100

leptin	TGFβ	low Glu	high Glu
100 ng/mL	5ng/mL	7mM	35mM

*, p<0.05 vs spontaneous

control	migration index(%)					
	1	2	3	mean	SD	SEM
0hr	0.00	0.00	0.00	0.00	0.0	0.00
24hr	8.73	15.22	15.22	13.06	3.7	2.16
48hr	18.15	29.14	29.14	25.47	6.3	3.66

TGFβ	migration index(%)					
	1	2	3	mean	SD	SEM
0hr	0.00	0.00	0.00	0.00	0.0	0.00
24hr	19.11	27.97	36.34	27.81	8.6	4.97
48hr	41.23	44.97	53.37	46.52	6.2	3.59

TGFβ+Lun	migration index(%)					
	1	2	3	mean	SD	SEM
0hr	0.00	0.00	0.00	0.00	0.0	0.00
24hr	15.91	36.18	24.93	25.67	10.2	5.86
48hr	33.74	43.74	53.60	43.69	9.9	5.73

leptin	migration index(%)					
	1	2	3	mean	SD	SEM
0hr	0.00	0.00	0.00	0.00	0.0	0.00
24hr	24.92	31.14	23.12	26.39	4.2	2.43
48hr	38.86	40.24	36.40	38.50	1.9	1.12

leptin+Lun	migration index(%)					
	1	2	3	mean	SD	SEM
0hr	0.00	0.00	0.00	0.00	0.0	0.00
24hr	25.22	25.99	13.10	21.44	7.2	4.18
48hr	40.32	33.50	30.46	34.76	5.0	2.91

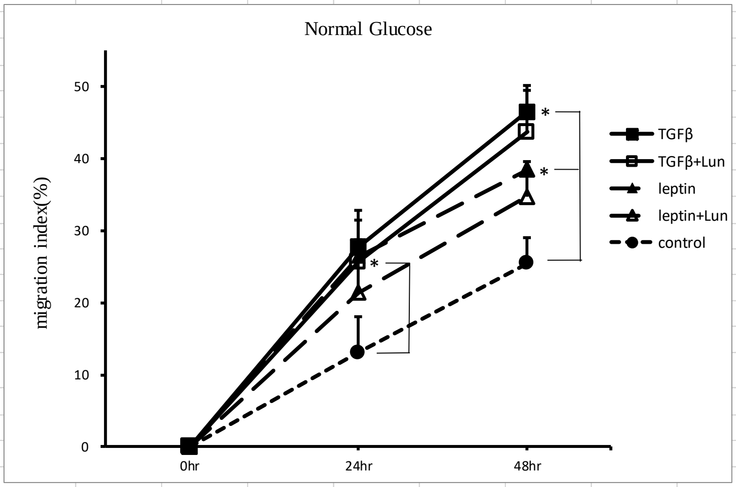

(B)

SPSS

TGFβ		migration index(%)						model	Lunasin
Time	treatment	1	2	3	mean	SD	SEM	p-value	p-value
0hr	control	0	0	0	0.00	0.0	0.00		
	TGFβ	0	0	0	0.00	0.0	0.00		
	TGFβ+Lun	0	0	0	0.00	0.0	0.00		
24hr	control	8.7	15.2	15.2	13.06	3.7	2.16		
	TGFβ	19.1	28.0	36.3	27.81	8.6	4.97	0.053	
	TGFβ+Lun	15.9	36.2	24.9	25.67	10.2	5.86	0.114	0.795
48hr	control	18.2	29.1	29.1	25.47	6.3	3.66		
	TGFβ	41.2	45.0	53.4	46.52	6.2	3.59	0.015	
	TGFβ+Lun	33.7	43.7	53.6	43.69	9.9	5.73	0.055	0.697

leptin		migration index(%)						model	Lunasin
Time	treatment	1	2	3	mean	SD	SEM	p-value	p-value
0hr	control	0	0	0	0.00	0.0	0.00		
	leptin	0	0	0	0.00	0.0	0.00		
	leptin+Lun	0	0	0	0.00	0.0	0.00		
24hr	control	8.7	15.2	15.2	13.06	3.7	2.16		
	leptin	24.9	31.1	23.1	26.39	4.2	2.43	0.015	
	leptin+Lun	25.2	26.0	13.1	21.44	7.2	4.18	0.149	0.363
48hr	control	18.2	29.1	29.1	25.47	6.3	3.66		
	leptin	38.9	40.2	36.4	38.50	1.9	1.12	0.027	
	letpin+Lun	40.3	33.5	30.5	34.76	5.0	2.91	0.118	0.297

(C)

area under the curve (AUC)

TGFβ		面積						model	Lunasin
Time	treatment	1	2	3	mean	SD	SEM	p-value	p-value
0hr-48hr	control	427	715	754	632	179	80		
	TGFβ	953	1211	1513	1226	280	125	0.036	
	TGFβ+Lun	787	1393	1241	1140	316	141	0.072	0.744

leptin		面積						model	Lunasin
Time	treatment	1	2	3	mean	SD	SEM	p-value	p-value
0hr-48hr	control	427	715	754	632	179	80		
	leptin	1064	1230	992	1095	122	55	0.021	
	leptin+Lun	1089	1026	680	932	220	99	0.141	0.323

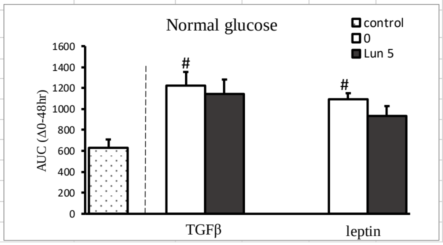

#, p<0.05 vs control

Figure 5.

(A)

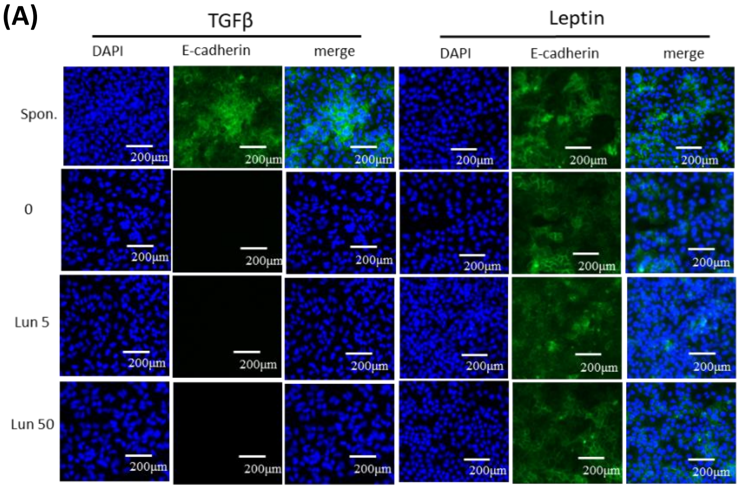

(B)

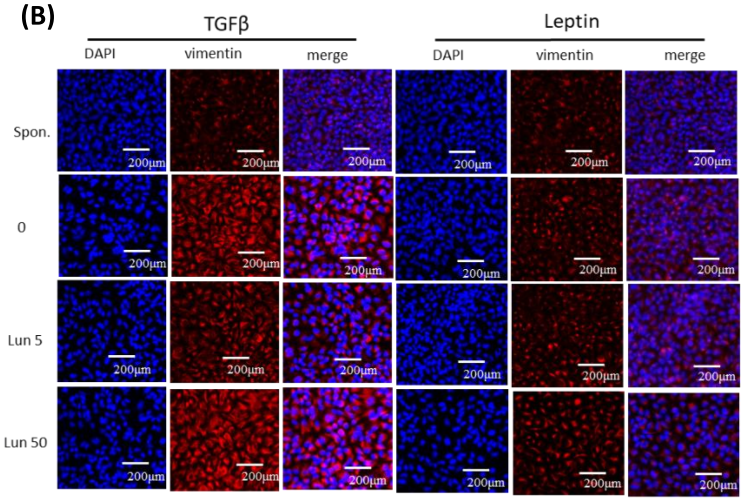

immunofluorescence

leptin	TGFβ
100 ng/mL	5ng/mL

對spontaneous的零點

(C)

E-cadherin		1	2	3	mean	SD	SEM	model p-value	Lunasin p-value
spontaneous	0	1.00	1.00	1.00	1.00	0.00	0.00		
TGFβ	0	0.20	0.43	0.03	0.22	0.20	0.11	0.002	
	Lun 5	0.56	0.53	0.10	0.40	0.26	0.15	0.055	0.402
	Lun 50	0.35	0.44	0.08	0.29	0.19	0.11	0.023	0.682
leptin	0	1.16	0.96	0.33	0.82	0.43	0.25	0.534	
	Lun 5	0.75	0.45	0.58	0.59	0.15	0.09	0.009	0.446
	Lun 50	0.99	0.56	0.42	0.65	0.30	0.17	0.181	0.622

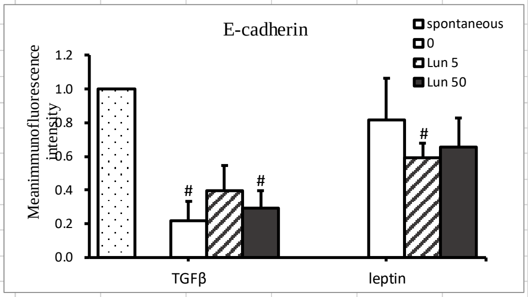

對spontaneous的零點

(D)

vimentin		1	2	3	mean	SD	SEM	model p-value	Lunasin p-value
spontaneous	0	1.00	1.00	1.00	1.00	0.00	0.00		
TGFβ	0	1.81	1.98	1.92	1.91	0.09	0.05	0.000059	
	Lun 5	1.29	1.79	1.81	1.63	0.29	0.17	0.065	0.198
	Lun 50	1.62	1.76	2.39	1.92	0.41	0.24	0.06	0.943
leptin	0	1.53	1.31	1.55	1.46	0.13	0.08	0.026	
	Lun 5	1.01	1.36	1.70	1.36	0.35	0.20	0.15	0.647
	Lun 50	1.49	1.10	1.40	1.33	0.20	0.12	0.108	0.403

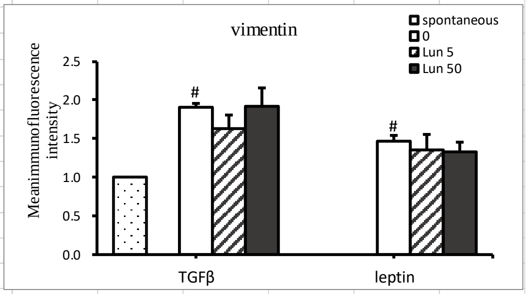

TGFβ
leptin

#, p<0.05 vs spontaneous

Figure 6.

in vivo lung cytokine- tissue homogenates

HF	high fat diet
HF-DL	HF-diet Lunasin

(C)

IL-6

22 wk

濃度÷ 蛋白質 (pg/ mg)

IL-6	1	2	3	4	5	6	mean	SD	SEM	p-value
HF	248.8	276.0	211.0	184.9	189.3	203.6	219	36.0	14.69	
HFL	220.0	179.9	182.4	199.8	179.2	162.6	187	19.9	8.12	0.089

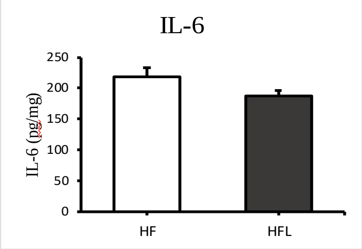

(D)

TNFα

22 wk

濃度÷ 蛋白質 (pg/ mg)

TNF-α	1	2	3	4	5	6	mean	SD	SEM	p-value
HF	19.1	24.4	20.4	22.6	20.9	21.9	22	1.8	0.75	
HFL	17.4	15.0	14.8	22.3	16.0	13.0	16	3.2	1.32	0.007

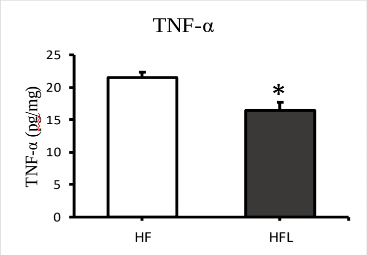

(E)

TGFβ

22 wk

濃度÷ 蛋白質 (pg/ mg)

TGF-β	1	2	3	4	5	6	mean	SD	SEM	p-value
HF	364.1	488.9	370.4	527.5	343.7	206.8	384	114.2	46.62	
HFL	258.8	164.3	217.4	182.3	220.5	248.7	215	36.7	14.97	0.006

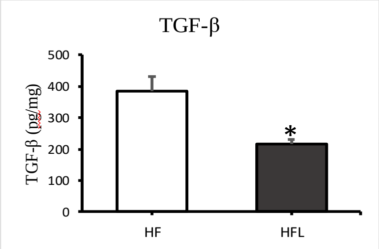

tissue culture lung cytokine-22wk

HF	high fat diet
HF-DL	HF-diet Lunasin

(F)

IL-6 missing data

濃度÷ 蛋白質 (ng/ x10 mg/mL)

IL-6	1	2	3	4	5	6	mean	SD	SEM	p-value
HF	84.4	91.4	39.9	38.0	35.7	9.1	50	31.7	12.94	
HFL	30.7	54.6	33.3	23.9	12.0		31	15.6	6.97	0.258
LPS HF	262.6	215.9	176.7	136.5	263.4	325.7	230	68.0	27.76	
LPS HFL	354.8	241.7	326.7	464.0	269.8		331	86.6	38.73	0.057

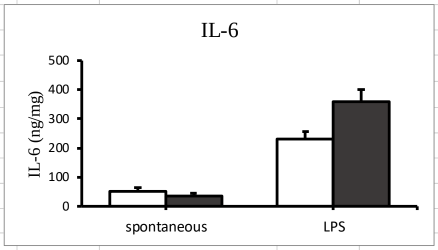

(G)

TNFα

濃度÷ 蛋白質 (ng/ x10 mg/mL)

TNFα	1	2	3	4	5	6	mean	SD	SEM	p-value
HF	5.9	7.7	2.7	6.1	6.4	5.0	5.6	1.7	0.68	
HFL	3.9	4.2	2.6	3.4	4.1		3.7	0.7	0.30	0.035
LPS HF	227.6	208.8	144.7	164.6	246.1	203.3	199	38.2	15.58	
LPS HFL	138.2	150.9	106.8	126.9	193.0		143	32.2	14.41	0.029

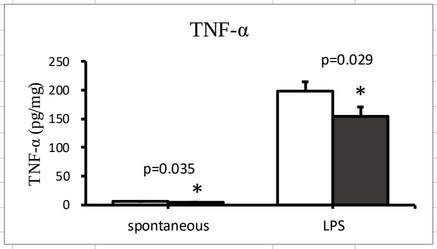

(H)

TGFβ

濃度÷ 蛋白質 (ng/ x10 mg/mL)

TGFβ	1	2	3	4	5	6	mean	SD	SEM	p-value
HF	125.2	149.7	112.8	89.3	84.1	133.9	116	25.6	10.45	
HFL	96.7	98.8	101.0	76.5	80.2		91	11.4	5.09	0.073
LPS HF	127.4	105.1	159.4	261.3	124.5	153.2	155	55.7	22.74	
LPS HFL	81.1	111.7	82.3	133.0	152.8		112	31.4	14.04	0.162

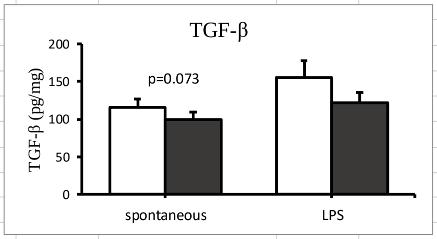

Figure 7.

Splenocytes cytokines

HF	igh fat diet	missing data
HF-DL	F-diet Lunasin	

spontaneous

IL-6	1	2	3	4	5	6	mean	SD	SEM	p-value
HF	38.9	35.2	48.9	42.4	44.9	56.1	44	7.4	3.04	
HF-DL	28.3	41.8		23.3	32.1	31.4	31	6.8	3.02	0.015

LPS

IL-6	1	2	3	4	5	6	mean	SD	SEM	p-value
HF	2254.7	1801.6	2356.3	2184.4	1832.8	2215.6	2108	232.5	94.90	
HF-DL	1903.1	2035.9	2447.7	1410.9	1754.7	1559.4	1852	369.0	150.65	0.182

spontaneous Non-detection

TNF-α	1	2	3	4	5	6	mean	SD	SEM	p-value
HF										
HF-DL										

LPS

TNF-α	1	2	3	4	5	6	mean	SD	SEM	p-value
HF	60.7	43.7	56.1	42.2	62.0	54.0	53	8.4	3.43	
HF-DL	55.9	55.6	54.3	40.1	46.4	78.3	55	13.0	5.29	0.763

spontaneous

IL-17a	1	2	3	4	5	6	mean	SD	SEM	p-value
HF	28.0	38.7	39.3	40.7	35.0	37.2	36	4.6	1.86	
HF-DL	39.1	43.6	37.6	30.5	35.6	35.6	37	4.3	1.77	0.847

LPS

IL-17a	1	2	3	4	5	6	mean	SD	SEM	p-value
HF	70.7	50.1	48.2	59.1	47.8	57.6	56	8.8	3.60	
HF-DL	59.3	80.3		48.9	52.5	51.7	59	12.7	5.70	0.662

spontaneous

IL-2	1	2	3	4	5	6	mean	SD	SEM	p-value
HF	0.6	1.4	1.5	1.3	1.2	1.3	1.2	0.3	0.13	
HF-DL	0.9	1.6	1.1	3.3	1.0	1.0	1.5	0.9	0.37	0.494

LPS

IL-2	1	2	3	4	5	6	mean	SD	SEM	p-value
HF	0.0	0.0	0.3	0.0	0.0	0.5	0	0.2	0.08	
HF-DL	0.0	0.2	0.0	0.0	0.0	0.0	0	0.1	0.03	0.304

spontaneous Non-detection

IFN-γ	1	2	3	4	5	6	mean	SD	SEM	p-value
HF	0.0	0.0	0.0	0.0	0.0	0.0	0	0.0	0.00	
HF-DL	0.0	0.0		0.0	0.0	0.0	0	0.0	0.00	

LPS

IFN-γ	1	2	3	4	5	6	mean	SD	SEM	p-value
HF	8.5	166.4		79.2	87.1	143.6	97	61.7	27.60	
HF-DL	192.6	238.5	228.8	205.4	263.7	350.6	247	56.7	23.16	0.002

spontaneous Non-detection

IL-4	1	2	3	4	5	6	mean	SD	SEM	p-value
HF	0.0	0.0	0.0	0.0	0.0	0.0	0	0.0	0.00	
HF-DL	0.0	0.0	0.0	0.0	0.0	0.0	0	0.0	0.00	

LPS Non-detection

IL-4	1	2	3	4	5	6	mean	SD	SEM	p-value
HF	0.0	0.0	0.0	0.0	0.0	0.0	0	0.0	0.00	
HF-DL	0.0	0.0	0.0	0.0	0.0	0.0	0	0.0	0.00	

ConA

IL-2/IL-4	1	2	3	4	5	6	mean	SD	SEM	p-value
HF	4.4	6.0	8.6	5.7	6.4	5.2	6	1.4	0.59	
HF-DL	12.8	20.1	11.3	19.2	12.4	23.4	17	5.0	2.04	0.003

ConA

IFN-γ/IL-4	1	2	3	4	5	6	mean	SD	SEM	p-value
HF	8.3	6.1	8.9	8.9	8.7	8.1	8	1.1	0.44	
HF-DL	9.4	10.1	16.5	9.8	7.4	10.1	11	3.1	1.26	0.104

ConA

IL-6	1	2	3	4	5	6	mean	SD	SEM	p-value
HF	842.2	510.2	521.9	518.0	373.4	424.2	532	163.6	66.77	
HF-DL	381.3	412.5		365.6	361.7	545.3	413	76.5	34.20	0.173

ConA

TNF-α	1	2	3	4	5	6	mean	SD	SEM	p-value
HF	21.4	18.6	18.3	34.9	35.4	34.1	27	8.5	3.46	
HF-DL	24.0	23.0	37.5	33.1	27.9	48.6	32	9.7	3.94	0.344

ConA

IL-17a	1	2	3	4	5	6	mean	SD	SEM	p-value
HF	1099	1033	1099	1053	1104	1017	1068	38.1	15.55	
HF-DL	1028	900	635	854	823	977	869	137.7	56.23	0.007

ConA

IL-2	1	2	3	4	5	6	mean	SD	SEM	p-value
HF	103.5	156.0	127.5	109.7	140.8	126.6	127	19.4	7.92	
HF-DL	181.8	171.1	135.8	253.8	133.7	303.6	197	68.2	27.84	0.055

ConA

IFN-γ	1	2	3	4	5	6	mean	SD	SEM	p-value
HF	195.7	158.8	131.9	170.9	191.6	197.8	174	25.9	10.58	
HF-DL	133.3	86.1	198.8	130.2	80.2	131.9	127	42.7	17.41	0.041

ConA

IL-4	1	2	3	4	5	6	mean	SD	SEM	p-value
HF	23.5	26.1	14.7	19.2	22.0	24.4	22	4.1	1.68	
HF-DL	14.2	8.5	12.0	13.2	10.8	13.0	12	2.1	0.84	<0.001

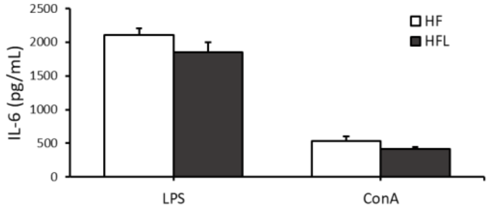

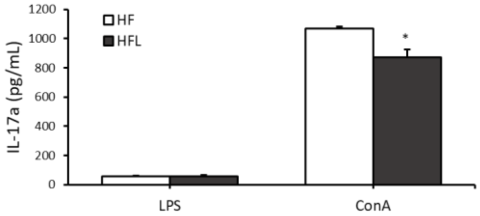

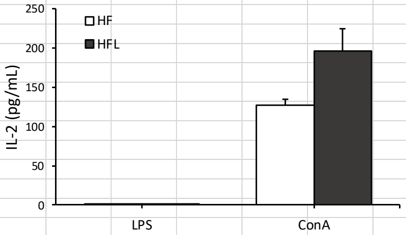

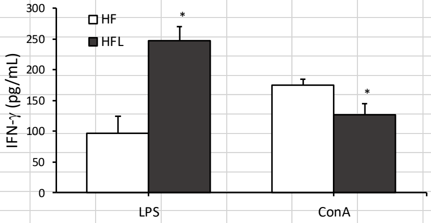

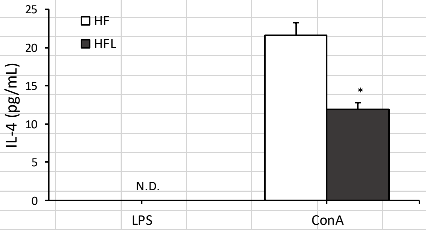

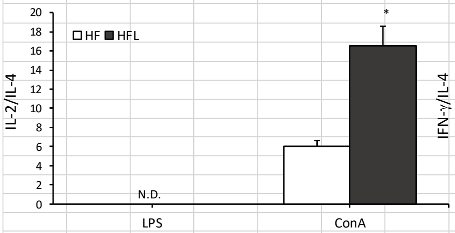

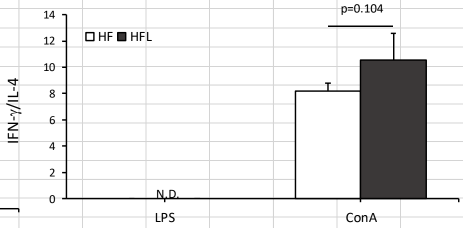

Supplement: Supplementary file 3 [file Supplementary_file_3.pdf]
